# Flexible Thermoelectric Wearable Architecture for Wireless Continuous Physiological Monitoring

**DOI:** 10.1021/acsami.4c02467

**Published:** 2024-07-09

**Authors:** Maria Sattar, Yoon Jae Lee, Hyeonseok Kim, Michael Adams, Matthew Guess, Juhyeon Kim, Ira Soltis, Taewoog Kang, Hojoong Kim, Jimin Lee, Hodam Kim, Shannon Yee, Woon-Hong Yeo

**Affiliations:** † George W. Woodruff School of Mechanical Engineering, Georgia Institute of Technology, Atlanta, Georgia 30332, United States; ‡ Wearable Intelligent Systems and Healthcare Center (WISH Center) at Institute for Matter and Systems, Georgia Institute of Technology, Atlanta, Georgia 30332, United States; § School of Electrical and Computer Engineering, College of Engineering, Georgia Institute of Technology, Atlanta, Georgia 30332, United States; ∥ Wallace H. Coulter Department of Biomedical Engineering, Georgia Tech and Emory University School of Medicine, Atlanta, Georgia 30332, United States; ⊥ Parker H. Petit Institute for Bioengineering and Biosciences, Institute for Robotics and Intelligent Machines, Georgia Institute of Technology, Atlanta, Georgia 30332, United States

**Keywords:** carbon nanotubes, energy harvesting, flexible, human body heat, human physiological signals, thermoelectric, wearable, wireless

## Abstract

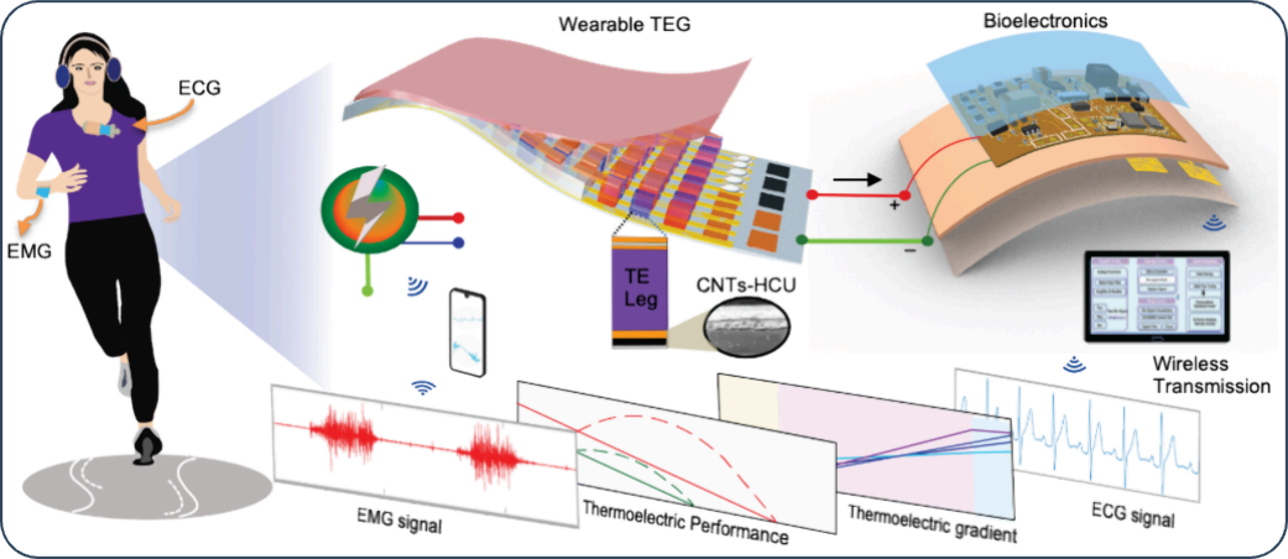

Continuous monitoring of physiological signals from the human body is critical in health monitoring, disease diagnosis, and therapeutics. Despite the needs, the existing wearable medical devices rely on either bulky wired systems or battery-powered devices needing frequent recharging. Here, we introduce a wearable, self-powered, thermoelectric flexible system architecture for wireless portable monitoring of physiological signals without recharging batteries. This system harvests an exceptionally high open circuit voltage of 175–180 mV from the human body, powering the wireless wearable bioelectronics to detect electrophysiological signals on the skin continuously. The thermoelectric system shows long-term stability in performance for 7 days with stable power management. Integrating screen printing, laser micromachining, and soft packaging technologies enables a multilayered, soft, wearable device to be mounted on any body part. The demonstration of the self-sustainable wearable system for detecting electromyograms and electrocardiograms captures the potential of the platform technology to offer various opportunities for continuous monitoring of biosignals, remote health monitoring, and automated disease diagnosis.

## 1 Introduction

Wearable health monitoring technology has transformed intelligent sensing systems to detect abnormalities with high efficiency.^1−5^ These systems precisely measure various health vitals like electrocardiograms (ECG), electromyograms (EMG), biomarker analysis, heart rate variability (HRV), organ dysfunction, nutritional malfunction, and emotional imbalances.^6−14^ These systems currently rely on lithium-ion batteries, which are lightweight and easily integrated but face limitations in fast charging and safety risks due to overcharging and overheating.^15−18^ In certain instances, overcharging and overheating of battery electrolytes pose the risk of flammability, and the corrosive electrolyte salts augment the danger of explosion.^15,19^ Self-powered energy harvesting technologies such as active energy systems or passive energy systems have become popular for powering wireless health monitoring systems reliably and safely.^20−23^ Thermoelectric generators (TEGs) directly convert heat into electricity through the Seebeck effect, providing a continuous power source independent of external factors like light intensity or body movements.^24^ The main advantage of TEG is its DC output voltage, which makes it easily integrated with the biosensor applications.^25^ The reliable and continuous provision of body heat makes thermoelectric technology a self-reliable energy source independent of electromagnetic waves, light intensity, metabolic rate, and body movements.^19,21,24,26,27^

Additionally, thermoelectric generators offer continuous power without maintenance and outperform other energy-harvesting technologies.^28^ Acknowledging its advantages, researchers stress the need to tackle challenges such as low power density and performance fluctuations due to temperature variations between the human body and the environment.^1,24,29−31^ Yet, none have achieved uninterrupted, long-term signal monitoring. Even if batteries meet long-term power needs, sudden load discharges shorten their lifespan, enlightening the careful use of energy sources, storage, and supply resources such as supercapacitors, batteries, and ultracapacitors to meet the power demand of load from milliseconds to hours.^32,33^ Extensive research has focused on enhancing the output power of wearable TEGs.^34−40^ However, miniaturized TEGs still struggle to deliver sufficient power for long-term health monitoring, whereas larger TEGs boast higher output power levels. For the sustainable performance of TEG, a higher thermal gradient across TE legs requires continuous and directional heat transfer with minimal thermal losses.^41^

Here, we introduce a body-heat-powered wearable health monitoring system enabling continuous physiological signal monitoring without interruptions. This self-recharging thermoelectric battery system operates independently, eliminating the need for intermittent recharging or battery replacements during diagnostics. Our approach focuses on enhancing the output power of wearable TEGs and ensuring thermal gradient stability for a fully portable, intelligent, and self-sustaining health monitoring system. Instead of solely improving thermal conductivity and heat transfer, we introduce a heat conduction unit (HCU) to increase the power density of the wearable TEGs for long-term monitoring. The HCU, printed only to the thermoelectric area, enhances heat transfer and reduces the form factor of wearable TEG systems. The HCU introduces an innovative pairing of carbon nanotubes with copper. We present a tailored communication protocol for wirelessly operating low-power ECG and EMG biosensors.

## 2 Results and Discussion

### 2.1 Overview of a Flexible Thermoelectric Wearable Device

**Figure 1:**
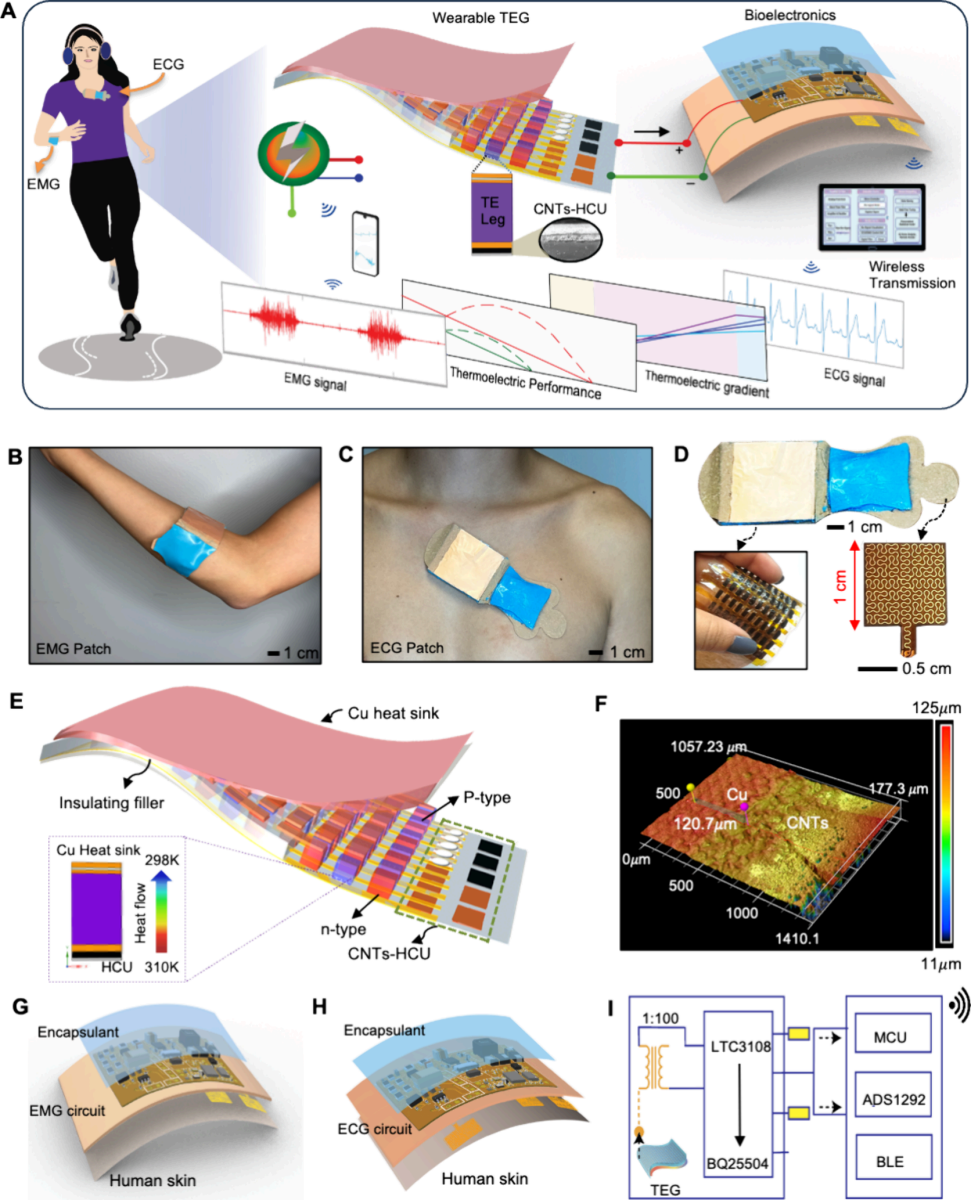
Overview of a flexible thermoelectric wearable device for wireless multimodal physiological monitoring. (A) Schematic illustration of a wearable system that shows the integration of human body heat-charged biosensor system, signal processing, and wireless acquisition of ECG and EMG signals. (B) Photo of a device mounted on the forearm to detect EMG signals. (C) Photo of a device mounted on the chest to detect ECG signals. (D) Photo of the device in panels B and C, integrating HCU and skin-like electrodes. (E) 3D cross-sectional view of a flexible thermoelectric unit with HCU and heat-insulating filler. (F) Surface profile image of screen-printed HCU on the Beyolex substrate: the bottom layer is CNTs, and the top layer is Cu. (G, H) Illustration of a multilayered EMG device (G) and ECG device (H) with different electrode arrangements. (I) System diagram including the step-up voltage circuit, power management circuit, and BLE-based wireless signal monitoring with an external device.

A self-sustainable wearable device is introduced in this work to address the vital challenges in wireless health monitoring. Figure 1A presents an overview of a flexible thermoelectric device that can measure continuous human physiological signals, including ECG, HRV, and EMG, in real time. The all-in-one wearable patch system is self-rechargeable by a high-performance carbon nanotube (CNT)-based TEG and uses Bluetooth to transmit the acquired data to personalized devices. To ensure the device’s performance, we optimized design parameters, such as the height of thermoelectric legs, thickness of the heat transfer layer at the receiving end, thermal conductivity of encapsulation, and conformal contact with human skin. Photos in Figure 1B,C show examples of wearable devices for detecting physiological signals from different body parts: one on the forearm for detecting EMG and the other on the chest for detecting ECG and HRV. The TEG device converts human body heat to electrical energy for measuring the target signals without recharging batteries and the support of wired devices.^42^ The wearable patch has two core components: a flexible TEG to power the system and an array of electrodes to detect biosignals (Figure 1D). Figure 1E shows the 3D cross-sectional view of a TEG, including four components: (1) CNT-based HCU, (2) thermoelectric component, (3) electrical conducting layer, and (4) top heat rejection layer. Through a set of experiments, these components were optimized thermally, mechanically, and electrically to ensure the reliable fabrication of TEGs. A stretchable material, Beyolex, has been used as the outer packaging layer because of its superior mechanical performance.^43^ The CNT film is a highly thermally conductive and heat-capacity layer to enhance heat transfer. CNTs own dual functionality of high thermal and electrical conductivity; to make them only thermally conductive, a bypass/buffer layer is a prerequisite.^44,45^ A screen-printed nano copper (Cu) layer over the CNT layer works as an electrical insulation because we only heat sintered the Cu film to remove the binder contents and build a robust interface. In addition, a laser-micromachined Cu sheet in a rectangular patterned electrode was chosen because of the enhanced conductivity and mechanical stability.

Furthermore, the Cu heat sink, which is thermally conductive, could improve heat rejection to maintain the thermal gradient inside the thermoelectric section. Figure 1F shows the surface profile of screen-printed CNT-HCU layers. This image captures intimate adhesion between CNT and Cu layers, demonstrating reliable fabrication with screen-printing techniques. We integrated two types of bioelectronic devices with the fabricated TEG, targeting EMG (Figure 1G) and ECG (Figure 1H) signals, respectively. We adopt a top-mounting approach of electrodes and circuits to reduce the device’s form factor and improve wireless signal transmission. Figure 1I shows the system diagram, including the step-up voltage circuit, a power management component, and BLE-based wireless circuit. The power management circuit processes the TEG output power to charge a 100 mF, 5.5 V polymer supercapacitor fully. Then, it charges a small lithium polymer battery for prolonged operation of the biosensor system to continuously measure physiological signals. Detected biosignals are wirelessly transmitted to a cloud system for data processing and classifications.

### 2.2 Development of an HCU-Embedded Flexible TEG

**Figure 2:**
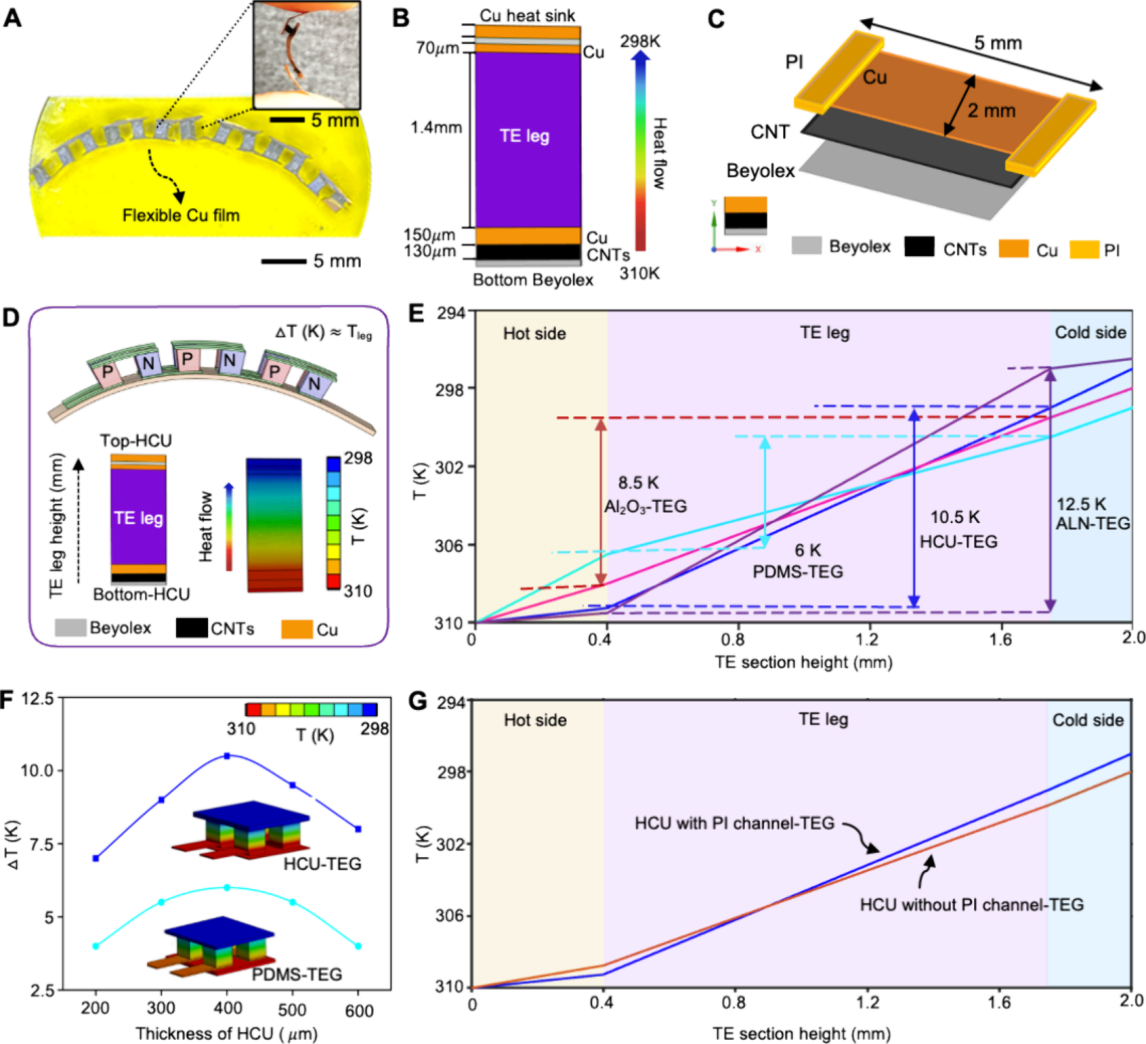
HCU-embedded TEG. (A) Side-view photo of a flexible TE unit showing a flexible Cu film as electrode. The inset shows a free-standing thermoelectric pair soldered with the Cu sheet. (B) Cross-section illustration of a TE leg with HCU. The vertical arrow indicates the direction of heat flow for the thermoelectric process. (C) Illustration showing the HCU materials and dimensions. (D) FEA result of the TE leg showing how the thermal gradient changes across the leg with HCU. (E) Characterization of different substrates to estimate thermal gradient variation corresponding to the thermal conductivity of substrates. (F) FEA result of the effect of HCU thickness with a PDMS substrate and an HCU with Beyolex. (G) Temperature distribution across TE leg with and without the Cu heat sink substrate.

An effective thermal gradient across thermoelectric (TE) legs is necessary to achieve maximum output power from the TEG. The thermal gradient of the human body and ambient is more than 10 K. Thermoelectric power generation depends on the thermal gradient across the TE legs, resulting from the conductive heat transfer from the hot surface to TE legs, heat flow inside TE legs, and heat rejection at the cold side. Microthermal management reduces the parasitic thermal losses from conformal contacts and soldering joints and the thermal impedance mismatch between TE materials and heat sources, enhancing the output power,.^41,46^ Several approaches used filling materials to increase the thermal gradient across the TE legs; i.e., polydimethylsiloxane (PDMS) and Ecoflex were considered insulating filling materials because of their extremely low thermal conductivity to reduce the thermal loss from the sideways of TE legs.^42,47^ These materials provide thermal insulation to TE legs and mechanical stability to the wearable TEGs. However, these materials cannot provide sufficient thermal insulation when compared to air as a thermal insulation in commercial rigid TEGs. Hence, we designed the TE section to stabilize a flexible TEG’s external and internal thermal gradient (Δ*T*) to improve the power density. A free-standing Cu sheet was soldered to form a thermoelectric chain for the mechanical stability of TE legs, as shown in Figure 2A. It shows the durability and flexibility of flexible Cu electrodes, adding mechanical strength to the TE legs without additional filling materials. As shown in Figure 2B, HCU governs four thermal conductive processes: (1) heat receiving, (2) heat storage, (3) heat transfer, and (4) heat rejection to stabilize the thermal gradient for maximum performance of TEG. We utilized the selective heat transfer layer limited to the TE section to maximize the internal heat transfer.

Further, heat losses from HCU were controlled by insulating the sideways with polyimide (PI) to reduce the heat loss inside the TEG (Figure 2C). The thermal series model to calculate the thermal conductivity of the thermoelectric section is explained in Figure S1. In the proposed HCU, a strategic pairing of single-walled carbon nanotubes (SWCNTs) and Cu expedites heat conduction, improving the TE process. It is noteworthy that thermal simulations are necessary to study the thermal compatibility of materials with thermoelectric materials. Hence, the finite elemental analysis (FEA) was conducted to study temperature distribution across the cross-section of the TE legs for separate TE legs (Figure 2D). FEA simulation in Figure 2E compared the temperature distribution for four different substrates—(1) alumina substrate, (2) PDMS substrate, (3) HCU-TEG, and (4) Aluminum nitride (AlN)-TEG—at a 12 K temperature gradient, which was easily achievable in the ambient environment. All the parameters for the FEA study are listed in Table 1.

**Table 1:** Thermal Conductivity of Materials and Parameters for FEA Study

materials	thickness (μm)	thermal conductivity (W m^–^^1^ K^–1^)
bottom HCU		
human skin	-	0.187
Beyolex	50	-
CNT	120	120-130
Cu	70	0.4
TE section		
p-type Bi2Te3	1400	1.3–1.4
n-type Bi2Te3	1400	1.81
top HCU		
Beyolex	50	0.35
3M Cu heat sink (*X*–*Y*/*Z*axis)	150	>270/0.80

In all cases, we assumed air as a thermal insulating filler. Although the encapsulation thickness was kept similar for all substrates (400 μm for the bottom layer and 300 μm for the top layer), the thermal gradient across the TE leg surprisingly varies for all substrates. FEA analysis shows that the HCU-TEG successfully established a significant thermal gradient of 10.5 K across the TE leg compared to the thermal performance of the rigid substrate of AlN, which established a thermal gradient of 12.5 K. The Al_2_O_3_-TEG is the commercial rigid TEG with no Cu heat sink. It used the alumina top layer as a heat sink. The thermal conductivity of Al_2_O_3_ is higher, but it has a lower heat capacity compared to CNTs. The novel organic–inorganic pairing is highly thermally conductive and has high heat capacity, holding a screen-printed CNT layer with a highly thermally conductive Cu heat sink. Al_2_O_3_ has a heat capacity of 0.88 kJ/kg·K;^48^ however, the CNT sheet has a heat capacity of 1.13 kJ/kg·K at room temperature. This makes a difference in the high heat transfer process in our innovative organic–inorganic pairing. Heat capacity is a necessary thermal property to maintain a reasonable gradient for continuous and sustainable power generation. AlN has high thermal conductivity of 140–180 W/m·K and a heat capacity of 0.74 kJ/kg·K.^49^ It has a lower heat capacity even than Al_2_O_3_ and it is rigid in nature. This makes our novel HCU-TEG with a Cu heat sink distinct with high thermal conductivity, high heat capacity, and flexibility with ease of fabrication.

The high thermal gradient is because of the high heat capacity and effective thermal conductivity of the CNT layer plus the well-established thermal interface of CNTs and Cu layers. However, the alumina and PDMS substrates established a lower thermal gradient of 8.5 and 6 K, respectively. It is because of the similar thermal conductivity layers on both sides of TEG and a significant thermal energy loss across the substrates. AlN offers high thermal gradient across the TE leg, but it is a rigid material with low heat capacity. For flexible TEG, the application of this material is difficult; however, Cu has a high thermal conductivity of 400 W/m·K, and the 3M heat sink is flexible as shown in Figure S2. The comparative analysis of the Cu heat sink with other possible flexible heat sinks is shown in Figure S3. The Cu heat sink successfully removes the heat from the cold side and results in the higher performance of TEG than Al foil and PDMS heat sinks.

We also performed FEA to optimize the thickness of the flexible substrate and compared the thermal performance of HCU thickness with PDMS thickness (Figure 2F). The HCU successfully maintains a higher thermal gradient than PDMS for varied thicknesses. This result is due to the CNT layer offering a dual function of heat-holding capability and high thermal conductivity. The experimental measurements of thermal gradient stability across the TE leg are given in Figure S4. Furthermore, PI insulation across HCU amplifies the effective heat transfer across the TE leg. The FEA of HCU with and without PI insulation shows that the insulation diverts and restricts the heat flow within the TE legs. HCU with PI insulation successfully established a higher thermal gradient (10 K without PI and 10.5 K with PI), as shown in Figure 2G. The overall design process depicts the flexible Cu sheet electrodes, the thermal performance of HCU, and the Cu heat sink, which collectively expedited the thermoelectric process and resulted in the high output performance of the TEG.

### 2.3 Fabrication and Characterization of a Wearable Flexible TEG

**Figure 3:**
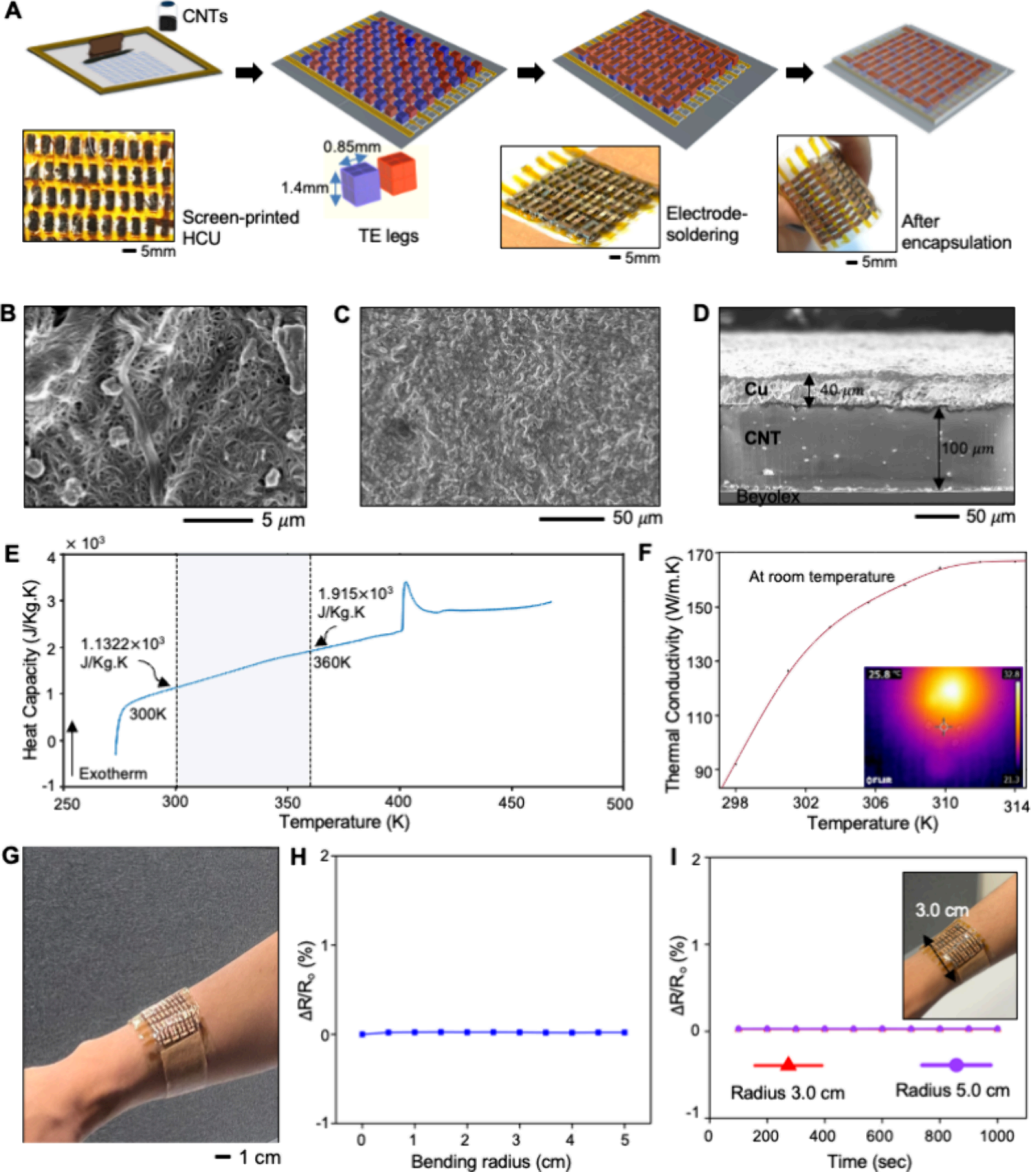
Fabrication and characterization of a wearable flexible TEG. (A) Illustration and photos showing the fabrication process of a flexible TEG, including (1) screen-printed HCU on a Beyolex substrate, (2) placement of TE legs on Cu electrodes, (3) soldering of TE sections at 170 °C for 5 min, and (4) encapsulation of the device. (B–D) SEM images of screen-printed CNTs (B), screen-printed Cu film (C), and cross-sectioned HCU with Cu and CNT films on a Beyolex substrate (D). (E) Specific heat capacity of the CNT film according to temperature (300–360 K). (F) Thermal conductivity of the CNT film. (G) Photo of a fabricated flexible TEG mounted on the skin. (H, I) Resistance change of the fabricated TEG at different bending radii (H) and the stability test for a prolonged duration (I).

The illustration and a series of photos in Figure 3A summarize the fabrication process of a flexible TEG. As the first step, HCU was screen-printed on the Beyolex substrate by following a five-step process: (1) synthesis of CNT ink, (2) screen-printing of CNT and Cu, (3) thermal sintering, (4) addition of the PI insulation channel, and (5) heat pressing of HCU. The detailed synthesis process of the CNT ink is shown in Figure S5. During fabrication, printed CNT films were air-dried and then heat-treated at 70 °C followed by screen printing of Cu ink. The Cu film was dried at 90 °C for 10 min. Then, a 100 μm thick adhesive PI sheet was cut using a laser micromachining system to prepare 2 mm width and 7 cm length insulating channels. As the next step, the HCU was manually heat-pressed using a roller to strengthen the unit. Afterward, Cu sheet electrodes were placed above the HCU, and high-performance Bi_2_Te_3_ TE legs were soldered to establish electrical connections. After assembling the TEG, the bottom and top Beyolex layers were sealed, and a Cu heat sink was added over the top layer.

Additional details of the fabrication processes appear in Figures S5 and S6. Scanning electron microscopic (SEM) images in Figure 3B capture a well-dispersed CNT film without loose ends. The SEM image in Figure 3C shows a highly densified Cu film, which serves as an insulation layer for the bottom CNTs. The screen-printed CNT and Cu layers make an interlocked interface, as captured in Figure 3D. The heat capacity graph in Figure 3E shows that the CNT film can hold a significant amount of heat: 1.1322 × 10^3^ J/kg·K at 300 K and 1.915 × 10^3^ J/kg·K at 360 K. This result validates the CNT’s functionality in reducing the thermal loss by storing and releasing a valuable amount of heat to the TE section. The interface energy for enhancing the thermal conductivity of the CNT film is manipulated by interlocking the interface with the Cu thin layer.^50^ A graph in Figure 3F presents the high thermal conductivity of the CNT film, as reported for individual CNTs.^51−54^ The thermal conductivity values increase as the temperature rises to 315 K and take a half parabola shape, which are consistent with the results in ref (51), covering the typical operation range of a TEG on the human skin. The Raman spectral analysis shows fewer defects in the screen-printed CNT film, proving that the fabrication process did not affect the microstructure of CNTs^55^ (Figure S7). The mechanical stability of the fabricated flexible TEG is critical considering the direct mounting on different body parts with various motions and skin deformation. Figure 3G–I shows the experimental results of the device’s mechanical reliability at different bending radii during a prolonged operation. The measurement result of electrical resistance with mechanical bending from 0.5 to 5 cm shows negligible changes. Video S1 shows the real-time mechanical performance of the flexible TEG during bending and folding in different directions. The flexible device shows stability when mounted on a coffee mug and a soft flask (Figure S8).

### 2.4 Performance Validation of a Wearable TEG

**Figure 4:**
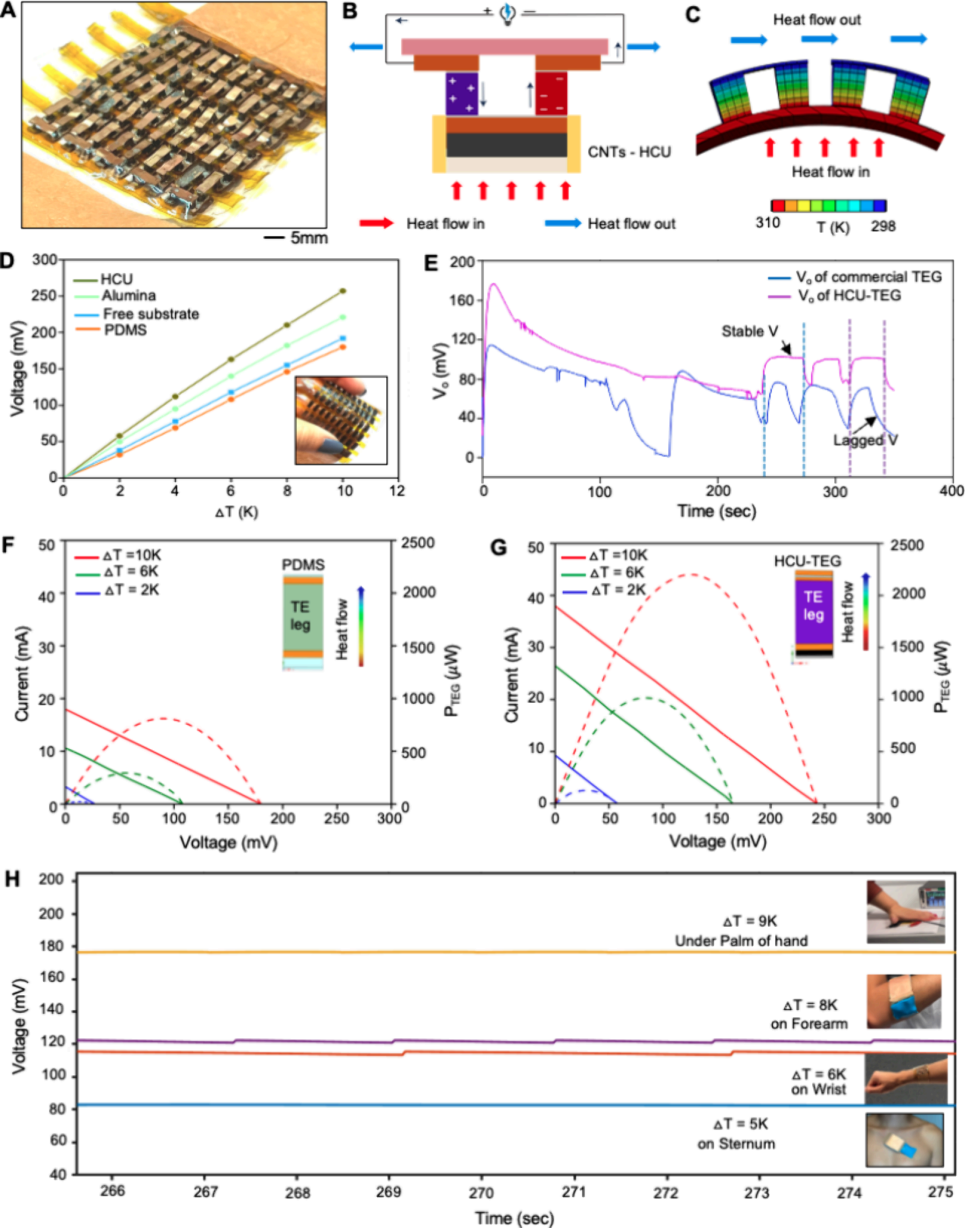
Performance validation of a wearable TEG. (A) Photo of a flexible wearable TEG. (B) Schematic illustration of the Seebeck effect on an HCU-embedded TEG. (C) Simulation result showing the thermal gradient of the TEG under bending. (D) Comparison of the open circuit voltage of flexible TEGs on different thermal conductive substrates for various temperatures. (E) Comparison of stable electrical performance between the flexible TEG and commercial TEG. (F) Measured output voltage, short circuit current, and corresponding output power of the flexible TEG on a PDMS substrate. (G) Measured output voltage, short circuit current, and corresponding output power of the flexible TEG on an HCU-embedded substrate. (H) Open circuit voltage of flexible TEGs mounted on different body parts, including the hand, forearm, wrist, and sternum.

Figure 4A shows a photo of a wearable TEG composed of 60 TE n–p pairs in a rectangular configuration. The illustration in Figure 4B explains the TE section’s Seebeck effect, which is the main TE principle defined as a thermoelectric body receiving heat toward its end. This heat converts into electrical energy, and electrons start to flow toward the cold end, which build an electric potential between the hot and cold end and result in Seebeck voltage.^21^ In our device, the CNTs-HCU receives heat, lets it pass through the TE legs, and then rejects it to the ambient environment through the top Cu heat sink. The FEA study in Figure 4C shows the Seebeck process and effective thermal gradient across TE legs. The Seebeck effect is calculated by the equation *S* = −Δ*V*/Δ*T*. The generation of open circuit voltage highly depends on the thermal gradient across the TE legs. Figure 4D summarizes the performance comparison of different substrates for generating the open circuit voltage under different thermal gradients. It is well-known that higher thermal conductivity increases the thermal gradient between TE legs and correspondingly increases the output performance. Our result shows TEG’s linear response, and the slope of the Seebeck voltage follows a linear trend by changing from 17.5 to 25 mV/K compared to the BN-doped PDMS substrate.^47^ This outcome indicates that HCU effectively harvests human body heat with negligible thermal loss even at a lower thermal gradient of 3 K. To validate the fabricated TEG’s long-term stability, we compared the performance with a commercial TEG, which uses alumina as a highly thermally conductive layer. The summarized data in Figure 4E show that the HCU-TEG outperforms the commercial one and generates an open circuit voltage of 175 mV at a thermal gradient of 9 K, the highest reported value. An example of the performance comparison appears in Video S2. Figure 4F captures that the maximum output power for the PDMS-TEG is 0.8 mW at Δ*T* of 10 K, whereas the HCU-TEG in Figure 4G shows the enhanced output performance: 2.3 mW at Δ*T* of 10 K, which is about 3 times higher than the PDMS one. We investigated the open circuit voltage of the wearable TEG mounted on different body parts, including the hand, forearm, wrist, and sternum. Utilizing a Beyolex sheet reduces the device’s overall weight, improving skin conformality and intimate contact with the skin for detecting high-quality physiological signals. On different body curvatures (Figure 4H), the wearable device generates open circuit voltages of 80, 118, 121, and 170–175 mV when mounted on the sternum, wrist, forearm, and under the palm, respectively. Based on the HCU TE architecture, our TEG generates the highest power density of 69 μW/cm^2^ at Δ*T* = 9 K among prior work reported to date. Table 2 compares this work’s materials and device performance with those of previously reported outcomes.

**Table 2:** Comparison of Materials and Device Performance between the Flexible Wearable TEG System in This Work and Prior Work

ref	flexibility (Δ*R*/*R*) %	measured signal	system material	material	TE architecture	power density (μW/cm^2^)
this work	0.01	EMG and ECG	organic–inorganic	SWCNTs/Cu/Bi–Te	HCU	69
(42)		ECG		Bi–Te	substrate free	13.8 at Δ*T* = 15K
(58)		CPU-temperature	inorganic	Bi–Te		
(76)	5		inorganic	Cu/Bi–Te	heat insulating layer	
(54)	1		organic	SWCNTS		
(50)			organic	CNP-CNT		0.0158
(46)			inorganic	Bi–Te/Ni	diffusion barrier layer	
(55)	2		inorganic	Bi–Sb–Te/Bi–Se–Te	directional heat layer	58.3
(40)	5		organic	SWCNTS/PEI		
(24)		humidity and temperature	inorganic	Bi–Te	polyimide base	3.5
(41)	50	temperature	inorganic	Bi–Te	soft heat conductors	6.96
(56)		temperature	inorganic	Bi–Te		
(8)		ECG	inorganic	Bi–Te	polymer-based flexible heat sink	38

### 2.5 Characterization of a TEG-Based Biosensing System

**Figure 5:**
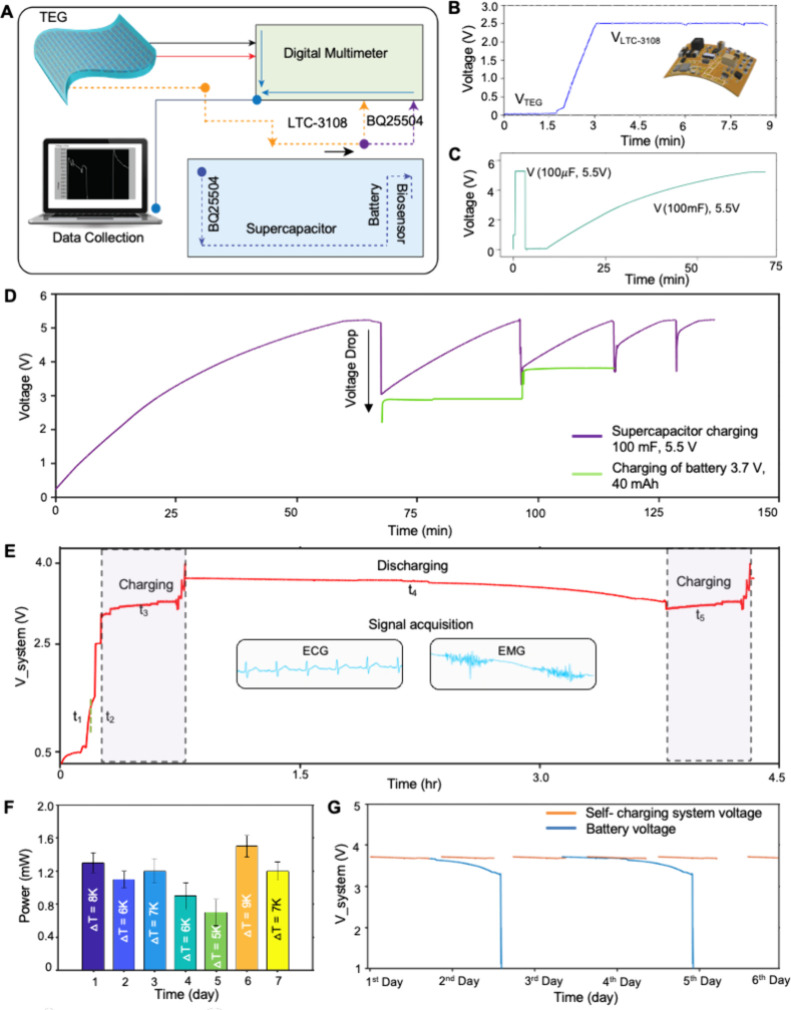
Characterization of a TEG-based biosensing system. (A) Illustration of the power management system for effectively utilizing human body heat to charge the supercapacitor and battery for biosensing. (B) Output voltage of the TEG boosted to 2.45 mV using LTC3108. (C) Charged supercapacitor using the boosted voltage from LTC3108. (D) Frequent supercapacitor charging with the flexible TEG and charging of a 40 mAh small rechargeable battery. (E) The entire system voltage from power generation, step up, and then power consumption via biosensing of ECG and EMG. (F) Long-term stability of the TEG system performance for 7 days. (G) Comparison of system voltage for 7 days between our device (self-charging system) and a battery-powered device. Our device has been running continuously and stable for a week.

To achieve the goal of a self-sustainable wearable system, the effective utilization of submilliwatt energy is necessary. We carefully audit this one-patch system’s energy to optimize the energy generation from wearable TEG, the biosensor power consumption, and the power required for wireless data transmission. In this study, physiological signal monitoring utilized ECG and EMG signals. For successful biosensor operation, a continuous supply of minimum required output voltage should be available to the system. In this study, we designed an effective power management system. As summarized in Figure 5A, this system effectively utilizes human body heat to charge the supercapacitor and battery for biosensing with four phases: (1) stable output of thermoelectric power, (2) load resistance matching, (3) boost-up circuit, and (4) energy storage/supply to the biosensor system. The detailed circuit diagram and board layout are available in Figures S9 and S10. After mounting and soldering all electronic components, the whole circuitry was encapsulated with an elastomer over an adhesive fabric. Figure 5B shows the continuous boost-up voltage of LTC3108 for 10 min to highlight the stable performance of the wearable TEG. For reliable load matching and maximum efficiency of the power management system, the input impedance was set at ∼8 Ω to match the internal resistance of TEG. This device sustains the boost-up operation of LTC3108 to power a 100 mF, 5.5 V supercapacitor (Figure 5C). The charging and discharging of the supercapacitor depend on the source of charging and the energy density of the end user system, whether it is a battery to which the supercapacitor discharges its volts or the biosensor circuit. The charge time of the supercapacitor is higher because of its higher capacitance. In Figure 5C, we show the charging of the 100 μF capacitor within a few seconds and then its quick discharge to a 100 mF supercapacitor. Then, the continuous charging of the 100 mF supercapacitor, which charges the battery, is shown in Figure 5D. The higher capacitance needs a higher charging rate, and the higher energy will be required to meet the energy density of the end user. The human body cannot maintain its temperature to a constant temperature. The fluctuation in the thermal gradient between the human body and ambient determines the charge time of the supercapacitor, which is why we used LTC3108 and BQ25504 to manage the charging of the supercapacitor and battery.^56^ The battery cannot be charged directly while powering the biosensor circuit. It needs to drain a certain amount of energy so that when the system is turned off, the supercapacitor discharges its power to the battery, and the battery will become available again at its full capacity in a time of need without being powered by the grid or other energy sources as shown in Figure 5E.

The discharge time of the supercapacitor is relatively less than the charging time because the battery has higher energy density than the supercapacitor. We use the supercapacitor to charge the battery or power the system because of its rapid discharge time.^57^ Álvarez et al. used a 50 mF supercapacitor to power wireless sensor nodes for a few minutes, and the supercapacitor discharged immediately from 5 to 2.5 V.^58^ Because it powered the sensor for a few minutes, they directly powered the system with a supercapacitor. However, we intend to perform long-term monitoring without any discontinuity, so we collected the energy in the supercapacitor and let it charge to at least 5 V at least so that it can meet the high energy density of a fully discharged battery and be able to charge a low-capacity battery for sustainable operation of biosensor.

The supercapacitor charges the battery continuously while being charged by the TEG, maintaining its voltage higher than the battery voltage. Figure 5F captures the long-term stability of the TEG system performance for 7 days when the device is mounted on the wrist. For a thermoelectric system, to ensure the efficient heat transfer, the thermal gradient of the thermoelectric system should follow this order: Δ*T*_supplied_ > Δ*T*_TEG_ > Δ*T*_TE_. It shows the Δ*T*_supplied_ (temperature difference between ambient and human body) from 5 to 9 K based on different room temperatures. For example, when there was 22 °C room temperature, the human body temperature was 31–32 °C, which built a Δ*T*_supplied_ of 9–10 K. When the room temperature was 25–27 °C, then the human body temperature at different positions was 29–33 °C, resulting in a temperature difference across TEG from 5 to 7 K. However, the temperature difference of TEG (Δ*T*_TEG_) and temperature difference of the TE leg (Δ*T*_TE_) are less than Δ*T*_supplied_ because of the thermal impedance mismatch at the interface of human skin and TEG, contact resistance thermal loss inside TEG, and joule heating effect. In our study, we calculated the ratio of Δ*T*_TEG_/Δ*T*_supplied_ = ∼64.5% which is higher than the literature reported values of ∼41^59^ and ∼26%.^31^ This ratio depends on the effective heat transfer between the top and bottom layers of the TEG device, which demands a rigorous approach for the selection of packaging material. Because the polymer has lower thermal conductivity as compared to CNTs and Cu heat sink, there will be less thermal gradient established in polymer-based TEGs. Figure S4 shows the thermal gradient of TEG in an ambient environment. The performance of TEG at different positions considering the temperature difference in an indoor environment is shown in Figure 4H. Frequent studies have reported high thermal gradients across TE legs; i.e., Lee et al. managed to get 8.5 K thermal gradient across 2 mm TE leg in a PDMS (*k* = 0.2 W/m·K at room temperature) encapsulation with soft heat conductors of AgNWs.^41^ In our study, we used a highly thermal conductive CNTs layer in HCU underneath the bottom electrode to keep the hot side temperature high enough so that the thermal gradient across TE leg was sustained, which helped us stabilize the thermoelectric performance for a longer time span than the commercial TEG. On the top side, the 3M Cu heat sink removes the heat at a faster rate, considering Cu's high thermal conductivity.

Figure 5G demonstrates the advantage of using our self-sustainable device compared to a battery-powered device. Our device has been running continuously and stable for a week without removing it from the skin or recharging a battery separately. We presented the sustainable performance of the flexible TEG in different atmospheric conditions i.e., indoor, humid, outdoor sunny, and wet conditions, as shown in Figure S11. The wearable flexible TEG successfully maintains its performance in all tested environments, as shown in Videos S3 and S4. Video S5 presents the real-time performance of this self-powered wearable health monitoring system.

### 2.6 TEG-Integrated Biosensing System for Measuring Physiological Signals

**Figure 6:**
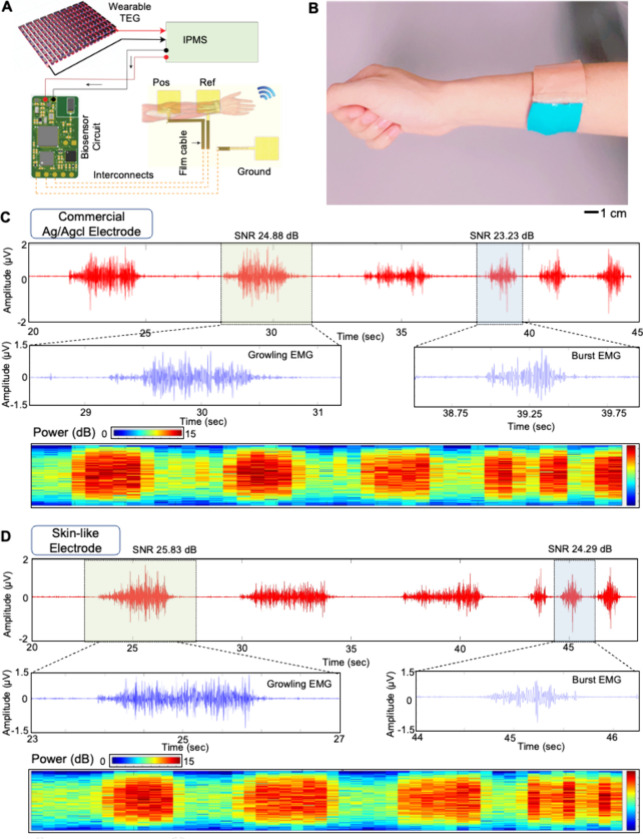
TEG-integrated biosensing system for measuring physiological signals. (A) Schematic illustration of a TEG-integrated biosensing system, including an array of electrodes and a wireless circuit to measure EMG and ECG signals. (B) Photo of the TEG-integrated biosensing system on the forearm for EMG signal acquisition. The skin-like electrodes make direct contact with the skin. (C) EMG signals measured by a set of commercial gel electrodes on the human forearm at a sampling frequency of 250 kHz during growling and bursting. (D) EMG signals measured by a set of skin-like dry electrodes on the forearm, showing similarities to the commercial one.

The body-heat-powered wearable device offers portable, continuous, wireless monitoring of physiological signals from the human skin. Figure 6A shows an example of a wearable biosensor system with an integrated wearable TEG. This device includes an intelligent power management system (IPMS), Bluetooth-low-energy microcontroller (BLE; nRF52832, Nordic), analog-to-digital converter (ADC; ADS1292, Texas Instrument), ceramic antenna, 1.7/3.3 V voltage regulator aligning with the power requirements of the microprocessor chipset, and an array of skin-like electrodes. The integrated device maintains a small form factor via multilayered stacking of components to provide skin conformality.^26,42^ The ADC, interfaced with electrodes, captures biosignals, subsequently transducing them from their analog forms into a digital format. The processed signals are then relayed to the microprocessor, which processes the transmission to a wireless receiver via a specialized low-power, high-bandwidth 2.4 GHz antenna. The details of the biosensor circuit layout appear in Figure S12. For the data reception and processing of physiological signals, this study employs the Flutter mobile application, chosen for its ability to handle complex data streams in real time. The total power consumption of our biosensing system is 1–2 mW at the start of a system^8,42,60,61^ and is then reduced to a few microwatts.^62,63^ The system uses a 3.7 V and 40 mAh battery and consumes 8 mW power for an operational time of 3.5 h without charging, and with charging, our system never runs out of power. ECG signals typically range from 0.05 to 100 Hz, whereas EMG signals can range from 20 Hz to 2 kHz. A bandpass filter accommodates both ranges to filter signals within this range selectively. The low-pass filter circuit and component are designed to attenuate frequencies above the highest frequency of interest. We designed a low-pass filter with a slightly higher than 2 kHz cutoff frequency to pass all relevant EMG and ECG signals. A cutoff frequency of around 2.5 kHz is used to preserve both signals. The high-pass filter circuit and component are carefully designed for removing DC offsets and very low-frequency noise, which are not part of the ECG or EMG signals of interest. Because ECG signals of interest start at about 0.05 Hz, a cutoff frequency of 0.02 hz, slightly below this range, filters out higher DC offsets and drifts. Amplification, converter, and digital signal acquisition are all common. The software has an internal algorithm to select modes of ECG/EMG.

This power requirement was a feasible index to be achieved by human body heat harvesting. Figure 6B shows the wearable biosensing patch mounted on the forearm to measure EMG signals. A photo in Figure S13 captures the location of electrodes integrated with a fabric substrate. A serpentine-patterned electrode (line width: 150 μm) makes direct contact with the skin. The mechanical performance of flexible circuits with fPCB electrodes is presented in Video S6. Flexible anisotropic conductive film (ACF) wires connect the electrode with the sensing circuit. EMG signals were measured on two muscle compartments on the forearm: anterior flexor and posterior extensor. The deep fascia of the forearm spreads around the musculature and is composed of the radius and ulna. The interior and exterior muscles cause muscle movements.^64^ Hence, the membrane electrodes cover the exterior and interior muscles to measure the EMG signals and form a parallelogram. We placed the recording electrode and reference electrode adjacent to each other at 0° and the ground electrode at 45° of the reference electrode so that the EMG electrode would locate 135° to the ground. Figure 6C captures the measurement outcomes from a commercial device to optimize the muscle location and compare the device’s performance with ours in Figure 6D. When mounted on the same location, our biosensing system shows a great signal-to-noise ratio (SNR) and fewer motion artifacts than the commercial one. The average EMG signal SNR was 25.83 and 24.29 dB during growling and bursting forearm muscles, respectively. The photo of the device for EMG monitoring is provided in Figure S14. Additional power analysis of EMG signal successfully detects the muscle movements during the events of growling and bursting. Video S7 shows an example of how this device measures EMG signals on the skin. The flowchart in Figure S15 shares a scenario that can use our wearable device for personalized portable at-home health monitoring using a tablet or a smartphone.

### 2.7 Measurement of ECG Data Using a Wearable Device

**Figure 7:**
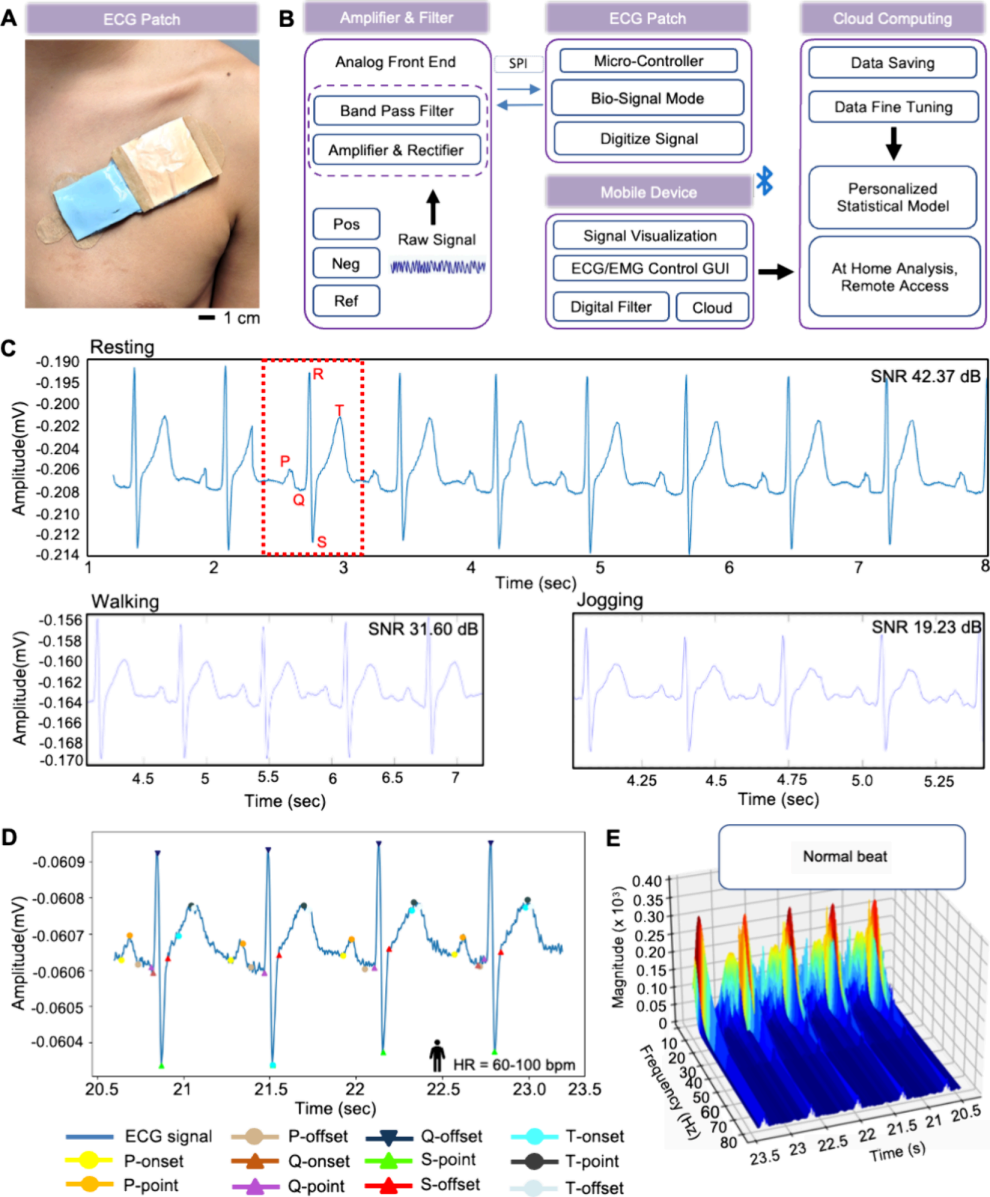
Measurement of ECG data using a wearable device. (A) Photo of an ECG patch mounted under the clavicle for wireless detection of ECG data. (B) Flowchart describing the data acquisition and multistep signal processing sequence. (C) Measured ECG data using the wearable patch in panel A showing clear detection of PQRST components during daily activities, including resting, walking, and jogging. (D) Detection of ECG PQRST component and HRV analysis using mathematical algorithms. (E) 3D plot showing normal heartbeat data.

Figure 7A shows a photo of a self-sustainable wearable health monitor for cardiac signal detection. Regarding the ECG measurement, the electrode location should be designed following the inflow and outflow of the heart blood between the right and left atrium and ventricle. The average size of the human heart is 12 × 8.5 × 6 cm.^65^ It is better if all electrodes are placed within these dimensions to acquire good signal quality. Therefore, all the electrodes for ECG monitoring were placed near the conduction system of electrical impulses from the heart muscle. The sinoatrial (SA) node, the point of origin of electrical impulses, is the heart’s natural pacemaker at the right atrium.^66^ Forming a scalene triangle near the SA node can be suitable for maximizing the electrical signals. Based on this geometry, electrodes are located on the base of the scalene triangle. A reference electrode is placed at the top of the scalene triangle in a way that the distance between the recording and reference electrodes is ∼3.2 cm and the distance between the reference and the ground is ∼3 cm. The flowchart in Figure 7B depicts the overview of the process of how we measure signals from the patch and deliver them to a mobile device and a cloud for data classification. The example in Video S8 shows the collection of ECG signals via the wearable patch compared with a commercial Ag/AgCl gel-electrode. The advantages of our device include consistent data acquisition with high SNR as shown in Figure 7C, even with walking and jogging conditions. All the ECG signals clearly capture the PQRST complex in health monitoring. Figure 7D shows the HRV statistics, measured from real-time ECG signals. A wavelet-based ECG delineator algorithm calculates the PR peak interval to monitor heart health.^67^ HRV is a measure of an interdependent regulatory system that helps to adapt to different environmental and psychological changes.^68^ Photos in Figure S16 show the placement of skin-like electrodes and the application of an ECG patch on the back. The ECG PQRST complex detector and HRV analysis were utilized with mathematical algorithms and temporal characteristics of fiducial points.^67,69^ The algorithm for detecting the PQRST complex in ECG signals included preprocessing stages to normalize and filter out the noise without distorting the signal’s crucial characteristics.^70^ Wavelet-based ECG delineator algorithms were employed to identify PQRST fiducial points accurately, particularly for class N heartbeats within the Arrhythmia Database.^67,71^ The core of the detection method utilizes a sliding window technique for identifying R-peaks, preserving the integrity of the QRS complex, and subsequently isolating the P and S waves. The approach accounts for common ECG signal artifacts by selectively enhancing non-QRS segments through a process involving Connecting Lines of Concave and Convex Points (CLCCP and CLCVP).^67,71^ The waveform’s fiducial points reduce the noise influence, enabling the algorithm to effectively differentiate between standard and aberrant wave patterns.^71^ The method detects fragmented QRS complexes, rendering it suitable for real-time applications and promising implications for enhanced heart rate variability analysis. For accurate calculation of HRV, we calculated PR, QRS, QT, and ST segments. We calculated the heart rate with this algorithm as 60–100 bpm at a PR interval of 0.12–0.20. All the measured parameters are summarized in Table 3. Wavelet algorithms indicated higher PR and lower QR interval led to higher HRV. The results of the algorithms are comparable with the findings of wavelet-based ECG delineator algorithms, which used PP, PR, and RR intervals to determine the HRV.^67^ Hence, our self-sustainable wearable health monitoring system can perform cardiac health monitoring by employing an intelligent wavelet-based ECG delineator (Figure 7E). At this stage, our system is designed for daily health monitoring of ECG/EMG signals in healthy individuals. We are working on expanding the system to diverse scenarios and human test plans to detect a broader range of abnormalities that our system effectively captures. This effort is directed toward improving our personal care applications and algorithms to these abnormalities, ultimately introducing a way for a new generation of wearable technology with TEG.

**Table 3:** Data Measured by the Wearable ECG Patch

target	value
heart rate	60–100 bpm
PR interval	0.12–0.20 s
QRS interval	0.06–0.10 s
QT interval	less than half of the R-R interval
ST segment	0.08

## 3 Conclusions

This paper reports on a self-sustainable, flexible, thermoelectric system for wireless physiological signal monitoring for a prolonged duration without changing batteries or using new devices. Integrating HCU-embedded TEG and low-power biosensor circuits fulfills the energy-saving requirement for continuous recording and detecting crucial physiological data in human health and/or performance monitoring. A comprehensive study of heat transfer materials, device design, thermomechanical architecture, efficient electronic integration, and less power communication protocol collectively addresses the challenges in developing a self-sustainable wearable device. The flexible device fabrication process presented in this work is easily scalable to reduce the device’s form factor without compromising performance. The demonstration of the wearable system in high-quality EMG and ECG detection, even at running conditions, captures this self-sustainable device’s potential in various clinical studies and at-home healthcare. This system that consumes microwatt-level energy is expected to be applicable for wireless long-term monitoring of critical biosignals in disease diagnosis and therapeutics.

## 4 Experimental Section

### Synthesis of the SWCNT Ink

CNTs in pristine form were purchased from TUBALL. Its specifications are given in Table 4. The CNT ink was synthesized using a wet chemical solution synthesis method. We first dissolved the CNT ink in deionized water with the assistance of an anionic surfactant in a proportion of 10:1. The solution was mixed on a magnetic stirring plate at 600 rpm. Afterward, CNTs were probe sonicated at 45 °C to disperse equally so that all agglomerates vanished. After that, the well-dispersed CNT solution was bath sonicated for 1 h, and then the CNT solution was again magnetically stirred at 60 °C for 3 h, resulting in a screen printable paste of CNT ink. Figure S5 shows the schematic representation of the ink synthesis process. The CNT ink was screen-printed on a Beyolex substrate (Panasonic, Japan). The Cu ink was purchased from Copprint Technologies. The commercial thermoelectric tiles to fabricate the thermoelectric module and a commercial thermoelectric generator SP1848-27145 for comparison were purchased from Comimark. For thermomechanical characterization of HCU, we performed scanning electron microscopic analysis, differential scanning calorimetry (DSC), and thermal conductivity analysis. Using a home-based thermoelectric setup, we calculated the electrical conductivity and the Seebeck coefficient of n-type and p-type Bi_2_Te_3_ thermoelectric legs. Based on manufacturer-provided thermoelectric properties and our homemade test setup, the thermoelectric parameters such as electrical conductivity (σ) and Seebeck coefficient (S) of n-type Bi_2_Te_3_ were 1250 S·cm^–1^ and 190 μV·K^–1^, and those for p-type were 1100 S·cm^–1^ and 201 μV·K^–1^. The following equation calculates the figure of merit ZT for n-type and p-type as 0.74 and 0.98 at 298 K, respectively:^21,30^*ZT* = (*S*^2^ × σ)*T*/*k*. The Cu sheet was used to laser cut the Cu electrodes for a 6 × 2 mm dimension. The surface and cross-sectional analysis of HCU was done using a microscope (SEM; Hitachi SU 8230).. A surface profiler was used to perform the surface profile study. A Raman spectrometer at 485 nm laser was used for the Raman spectrum analysis of the CNT ink. For designing the wearable system, the Altium PCB Designer designed a power management circuit, which was then ordered commercially for effective manufacturing. Schematics of the circuit are shown in Figure S9, and a list of electronics components for battery management and biosensor circuits is given in Tables S1 and S2. All components were soldered using the conventional heat gun and soldering iron at 180 °C. The FEA analysis was done using the Ansys simulation software. We built the geometry in the Ansys program and imported it to the Ansys mechanical workbench for thermoelectric simulation. All the simulations were done by setting a 12 K thermal gradient. The thermal conductivity of CNTs film was measured in-house by the self-heating method.^51,52^ We prepared CNT buckypaper, cut a sample of 1 × 1 cm CNT sheet, heated it by applying voltage from 0.5 to 2 V, and measured the corresponding current by using a high-precision digital multimeter (Keithley 7510). The temperature of the film was measured by using an infrared gun (IR). Then we used the analytical model to calculate the thermal conductivity of the CNT film,.^52^

**Table 4:** Key Parameters of SWCNTs^53^

parameter	SWCNTs
typical diameter	1–2 nm
typical length	up to 1 mm
aspect ratio	up to 10,000
elastic modulus	1000–3000 GPa
tensile strength	50–100 GPa
thermal conductivity at 300 K	3000–6000 W/mK

### Thermoelectric Test Setup

All the thermoelectric parameters were measured using a digital multimeter, and data were recorded using the MATLAB code and LabVIEW program. The Seebeck coefficient measurement used a homemade setup based on two Peltier thermoelectric modules with temperature sensors (Figure S17). One Peltier cooler module was used to heat the thermoelectric sample, and the other was used to remove the heat from the thermoelectric sample, establishing a thermal gradient. The change in voltage was recorded on a LabVIEW program, and then we used the general Seebeck coefficient formula to calculate the value. The same procedure was followed for Seebeck coefficient measurements of TE legs. All the experimental readings were measured at least three times. Then, all values were averaged. The efficiency of our TEG comes at 1.9–4.0% by using the equation
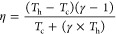
where *T*_h_ is the hot side temperature, *T*_c_ is the cold side temperature, and γ = (1 + *ZT*)1/2.^72^

### Mechanical Reliability Test

The mechanical stability was determined by estimating the change in resistance of the wearable TEG during bending at different radii. The wearable TEG was held from both sides with a clamp, and then Mark 10 was used to measure the resistance. The change in resistance was calculated by subtracting the measured values from the initial resistance value and dividing them by the initial resistance. A similar procedure for bending resistance analysis on the coffee mug, human body, and flask was followed. Figures S18–S20 show the real-time mechanical performance.

### Adhesive Fabric Preparation

For adhesive fabric preparation, parts A and B of Silbione (A-4717, Factor II) as an adhesive were manually mixed in 1:1 proportion for 5 min. Then, uncured Silbione was poured onto a plastic substrate and spin-coated at 1000 rpm for 60 s. Then, an adhesive fabric tape was placed carefully on top of Silbione, and the oven was baked at 65 °C for 30 min. After successfully curing Silbione on the back of the fabric tape, the sacrificial plastic substrate was removed to place electrodes on the cured Silbione.

### Flexible Electrode Preparation

The flexible printed circuit board (FPCB) electrodes were designed in AutoCAD and them imported to the PCB design software Altium Designer to make the PCB layout, and then we ordered it from commercial vendor PCBway to fabricate the fPCB electrodes. The design of the electrode follows the electrode design convention from ref (73). fPCB skin electrodes are gold electrodes deposited on a hydrophobic polyimide (PI) layer as a base that added stability to these electrodes, as shown in Figure S13C. Electrodes are placed on an adhesive tape made of breathable fabric with an additional Silbione layer, which offers the best contact with the skin. Silbione, a hydrophobic and washable polymer, offers stable performance in wet conditions.^74,75^

### Device Assembly

The wearable TEG was attached to a biosensor circuit to fabricate a self-sustainable wearable health monitoring system. The power management circuit was placed on top of the adhesive fabric with skin electrodes underneath. After soldering electronic components on all circuits, the skin electrodes were connected to the biosensor circuit using ACF wires and silver adhesive paint. Then, after drying the silver paint and confirming that the circuit was working, all the electronic components were encapsulated with an elastomeric layer (Ecoflex 00-30, Smooth-On). This material was also applied at the intersection of adhesive fabric and wearable TEG (Figure S16).

### Human Subject Study

The study involved multiple subjects, and it was conducted by following the approved IRB protocol (#H22289) at the Georgia Institute of Technology. Before the noninvasive study, all subjects agreed with the study procedures and provided signed consent forms.

## Data Availability

The data that support the findings of this study are available from the corresponding author upon reasonable request.

## Supporting Information Available

Thermal conductivity model for the estimation of thermoelectric process; thermal resistance in series model with thermal gradient across the thermoelectric legs; the camera images of the 3M Cu heat sink tape; the comparative analysis of output performance of the flexible TEG with flexible encapsulations, i.e., PDMS heat sink, Al foil heat sink, and Cu heat sink; the thermal gradient measurements across one TE pair; the synthesis of screen-printed CNTs ink for the fabrication of HCU; the camera images of the fabrication process of flex-WTEG; Raman spectrum analysis for the p-type and n-type SWCNT ink; the flexibility image of the wearable TEG on varied diameters; power management circuit and schematics of power management, the flexible PCB board for power management circuit, the camera images of flexible PCB board circuit, table of electronics components for biosensor and power management circuit; the flexible PCB board of biosensor circuit for the physiological signal acquisition; the performance of the flexible TEG with novel HCU in varied environments, i.e., outdoor sunny day, humid, and indoor (inset shows the focused view of voltage output); illustration of the layered biosensor device; camera image of the biosensor device; camera image of fPCB skin electrodes; camera image of the EMG device before encapsulation; proposed flowchart for personalized-at-home health monitoring; camera image of skin electrodes on the back of the adhesive tape; camera image of the ECG detection system on a human’s back; the testing setup including computer and digital multimeters for thermoelectric properties measurement; and the CNT film on a thermoelectric station; the real-time mechanical performance of the TEG device when bent, folded, and swung in different directions as shown in Video S1; the real-time bending performance of electrodes for various bending cycles; and the real-time performance of the TEG device in a wet environment (PDF)
Mechanical performance of the flexible TEG (Video S1) (MP4)
Performance comparison between our device and a commercial one (Video S2) (MP4)
Sustainable performance of TEG in a wet environment (Video S3) (MP4)
Comparison of TEG performance in different climates (Video S4) (MP4)
Self-powered wearable health monitoring system (Video S5) (MP4)
Mechanical performance of the flexible PCB (Video S6) (MP4)
Device that measures EMG signals (Video S7) (MP4)
Device that measures ECG signals compared to a commercial one (Video S8) (MP4)
