# Supplementary material for: Flexible Thermoelectric Wearable Architecture for Wireless Continuous Physiological Monitoring
Source: ACS Appl Mater Interfaces. 2024 Jul 9;16(29):37401–17. doi: 10.1021/acsami.4c02467 (PMC11284755; doi:10.1021/acsami.4c02467)
Supplement: Supplementary file 1 — am4c02467_si_001.pdf [file am4c02467_si_001.pdf]

Supporting Information

Flexible Thermoelectric Wearable Architecture for Wireless Continuous Physiological Monitoring

Maria Sattar[a,b,1], Yoon Jae Lee[b,c,1], Hyeonseok Kim[a,b], Michael Adams[a,b], Matthew Guess[a,b], Juhyeon Kim[b], Ira Soltis[a,b], Taewoog Kang[a,b], Hojoong Kim[a,b], Jimin Lee[a,b], Hodam Kim[a,b], Shannon Yee[a], and Woon-Hong Yeo[a,b,d,e]*

[a] George W. Woodruff School of Mechanical Engineering, Georgia Institute of Technology, Atlanta, GA, 30332 USA

[b] Wearable Intelligent Systems and Healthcare Center (WISH Center) at Institute for Matter and Systems, Georgia Institute of Technology, Atlanta, GA, 30332, USA

[c] School of Electrical and Computer Engineering, College of Engineering, Georgia Institute of Technology, Atlanta, GA, 30332, USA

[d] Wallace H. Coulter Department of Biomedical Engineering, Georgia Tech and Emory University School of Medicine, Atlanta, GA 30332, USA

[e] Parker H. Petit Institute for Bioengineering and Biosciences, Institute for Robotics and Intelligent Machines, Georgia Institute of Technology, Atlanta, GA, 30332 USA

[1] *These authors made equal contributions: co-first authors*

*Corresponding author email: whyeo@gatech.edu

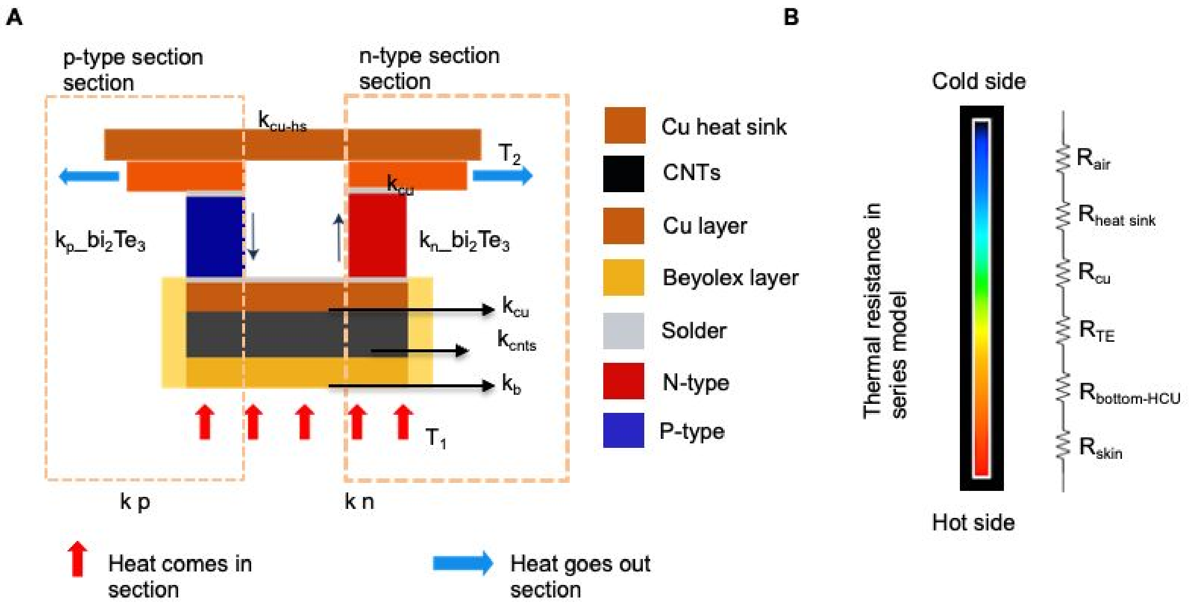

Figure S1: A) The cross-sectional view of HCU for the estimation of the thermoelectric process; B) Thermal resistance in series model with a thermal gradient across the thermoelectric legs.

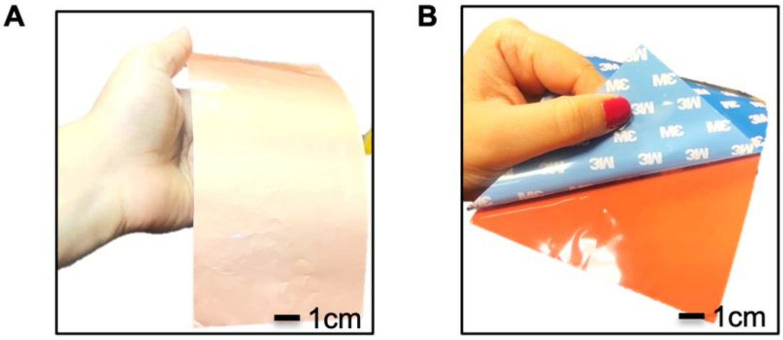

Figure S2: A) Top side of 3M tape for Cu heat sink in Figure S4; B) Back side of the tape.

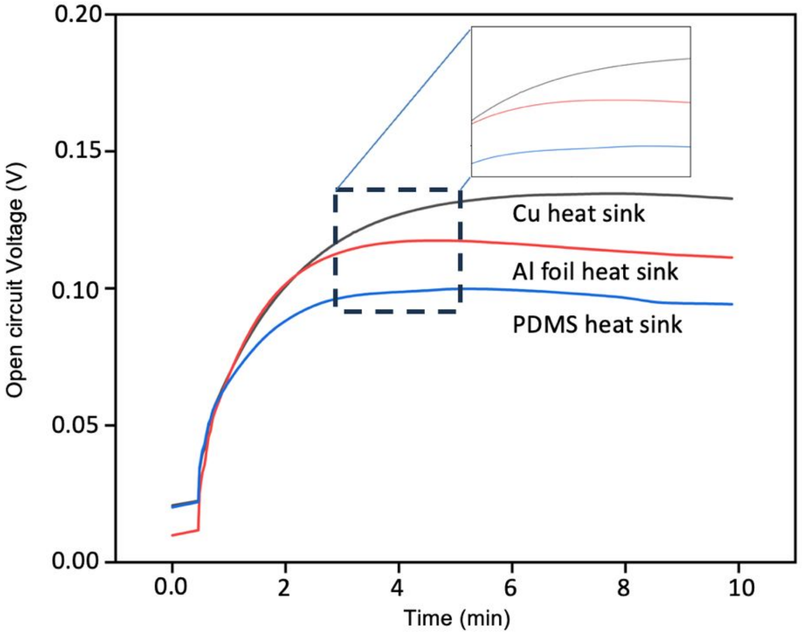

Figure S3: The comparative analysis of output performance of flexible TEG with flexible encapsulations, i.e., PDMS heat sink, Al foil heat sink, and Cu heat sink

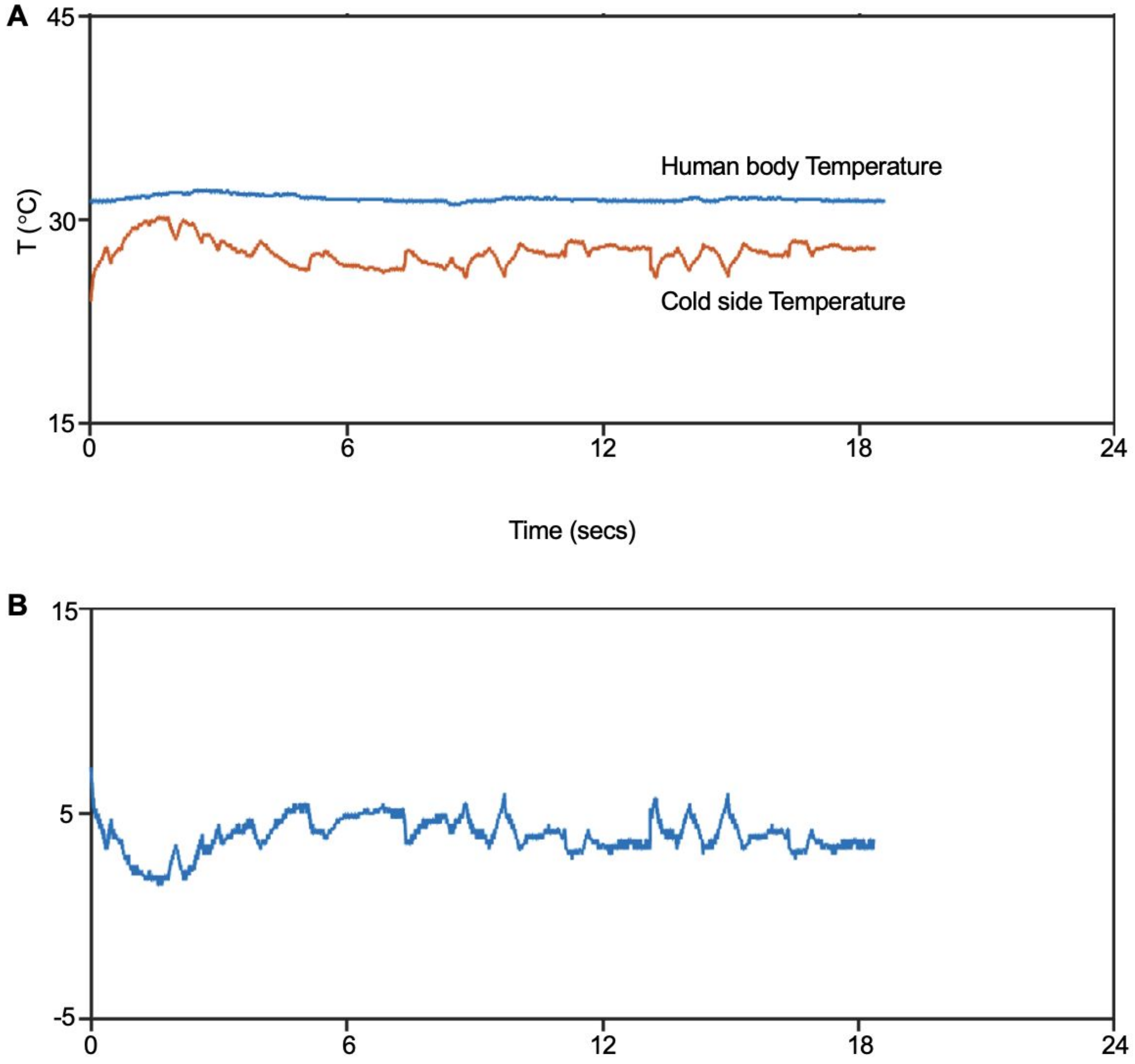

Figure S4: A) The temperature measurements at the top and bottom of the TEG device, B) The thermal gradient measurements of the TEG device 112 pairs

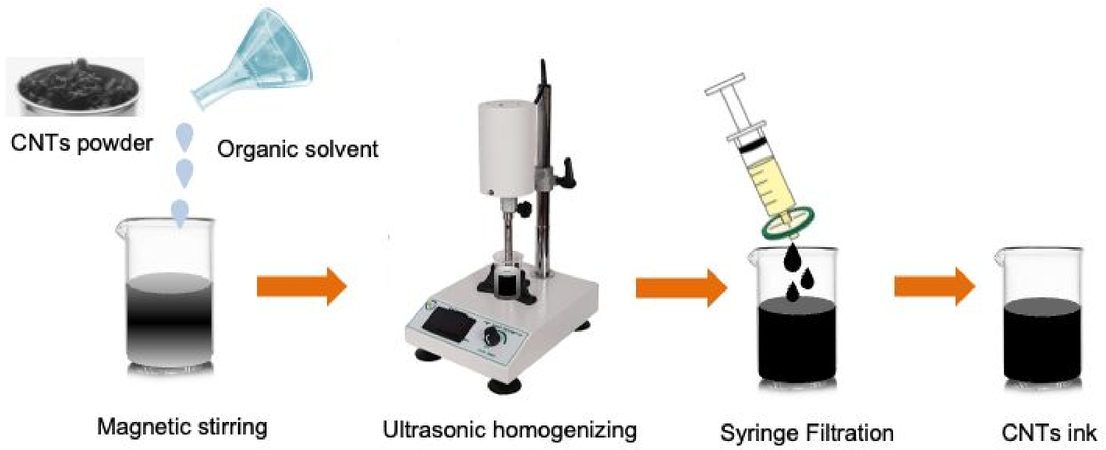

Figure S5: Synthesis of screen-printed CNT ink for the fabrication of HCU.

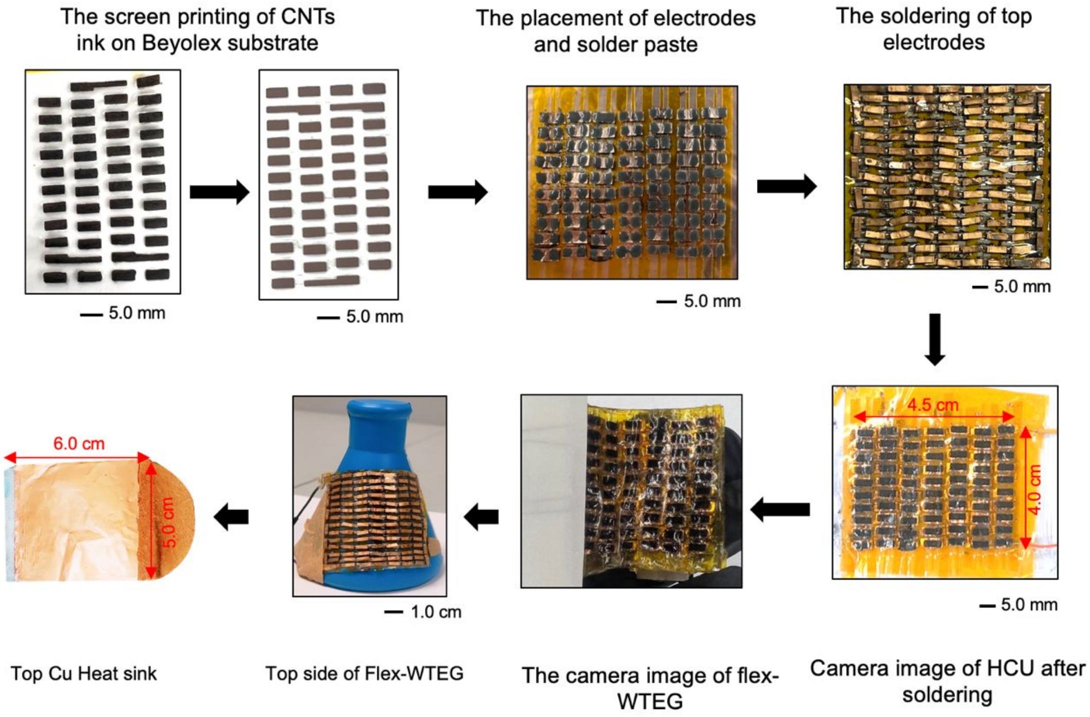

Figure S6: Fabrication process of a flexible wearable TEG.

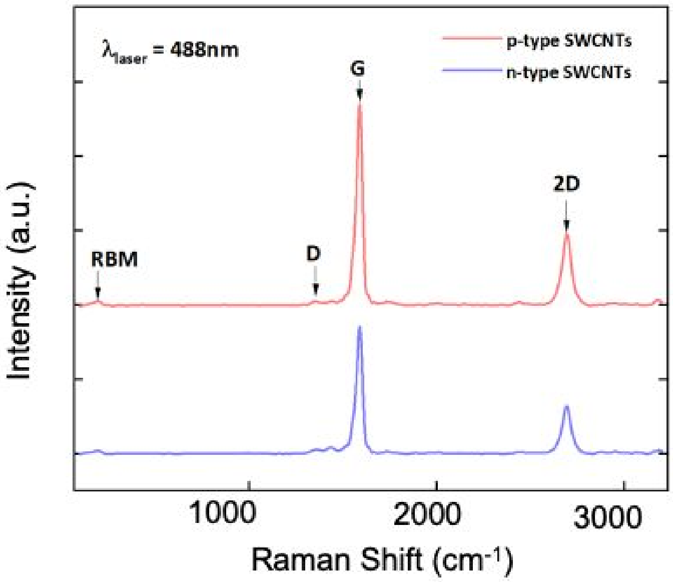

Figure S7: Raman spectrum analysis for the p-type and n-type CNTs ink.

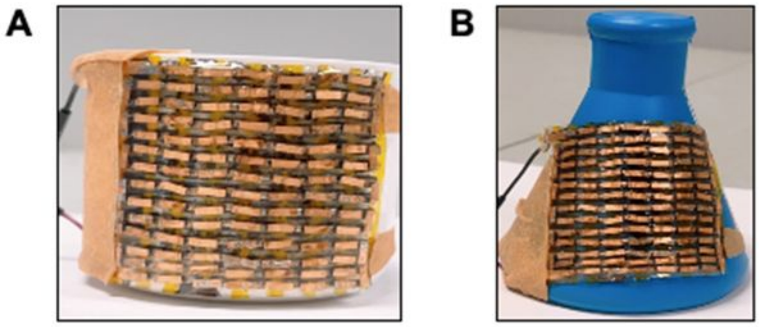

Figure S8: A) Flexible TEG on a coffee mug; B) The same device on a soft flask.

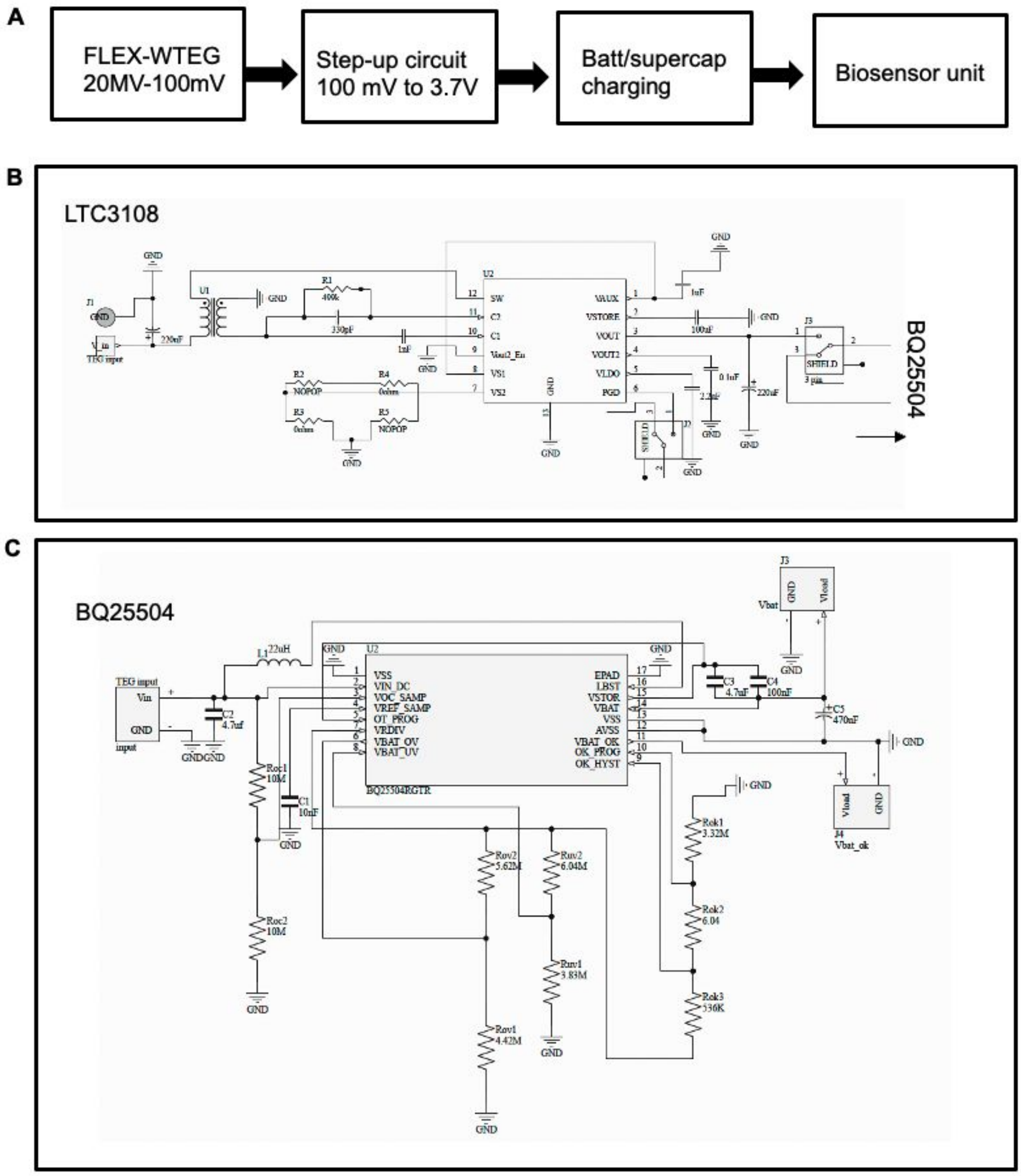

Figure S9: A) flow chart of a power management circuit, B) LTC3108 circuit, C) BQ25504 circuit.

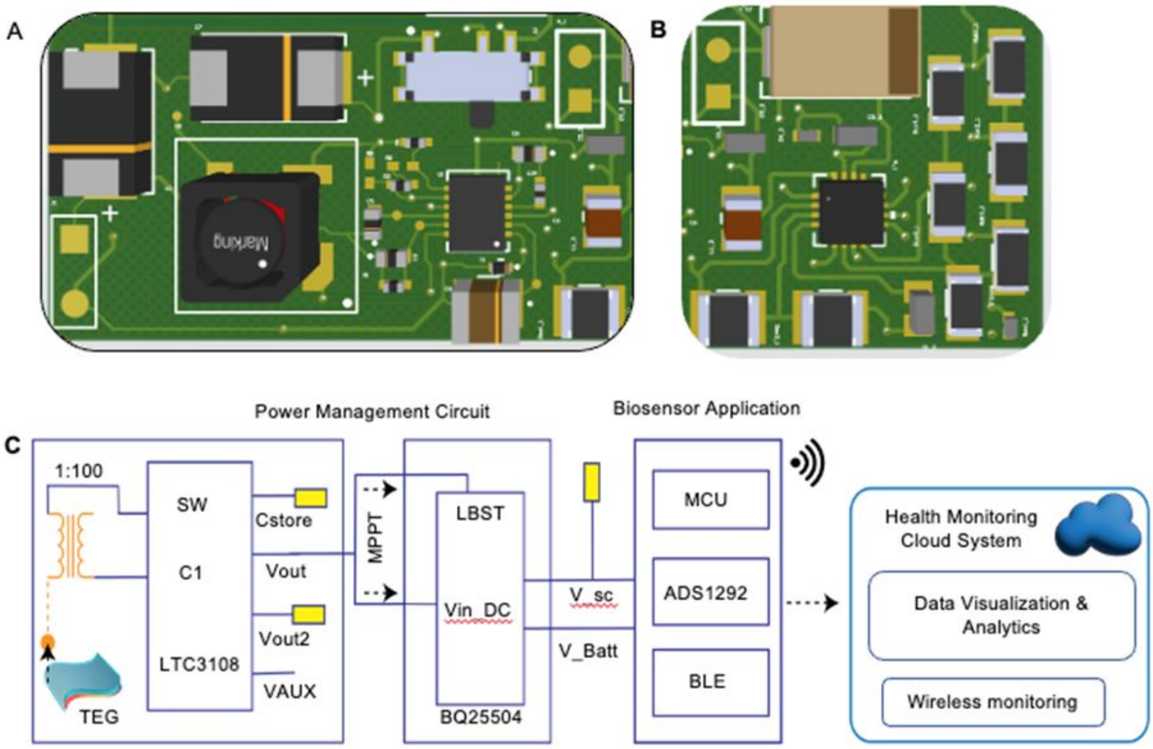

Figure S10: A) Step-up voltage circuit; B) Battery charge circuit; C) Flow chart of circuit integration for biosensing.

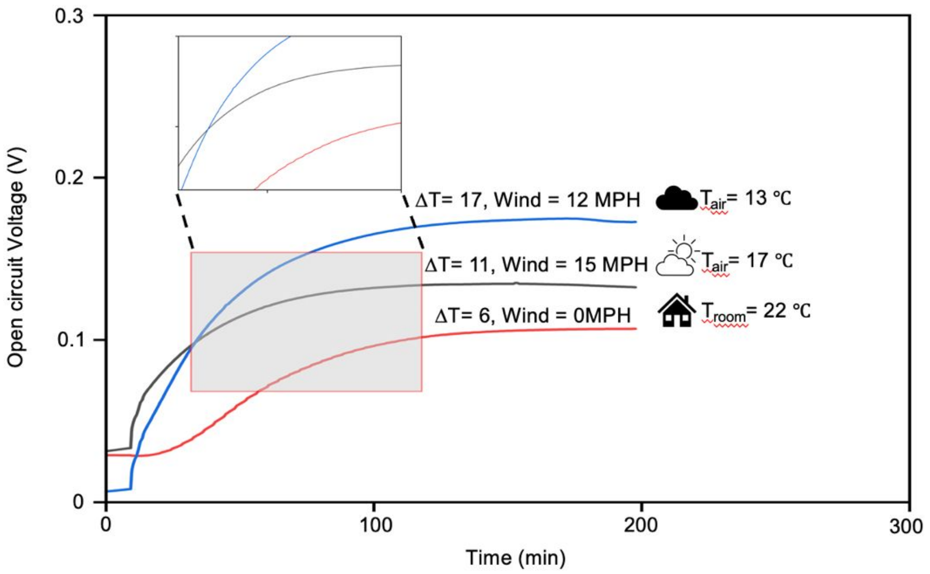

Figure S11: The performance of flexible TEG with novel HCU in varied environments, i.e., outdoor sunny days, humid, and indoor – inset, shows the focused view of voltage output.

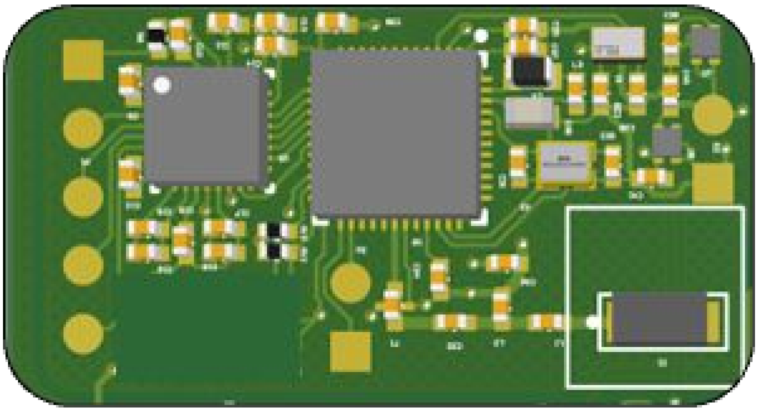

Figure S12: Biosensor circuit for the physiological signal acquisition.

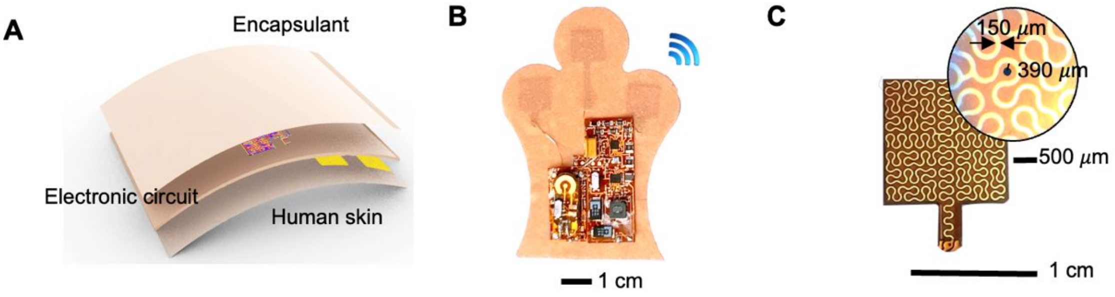

Figure S13: A) Illustration of the device; B) Photo of a fabricated device; C) Photo of a skin-like electrode. The inset shows serpentine patterns with a width of 150 μm.

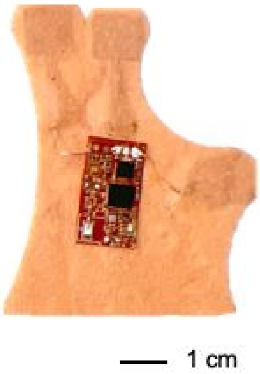

Figure S14: Photo of a device for EMG monitoring with an array of electrodes.

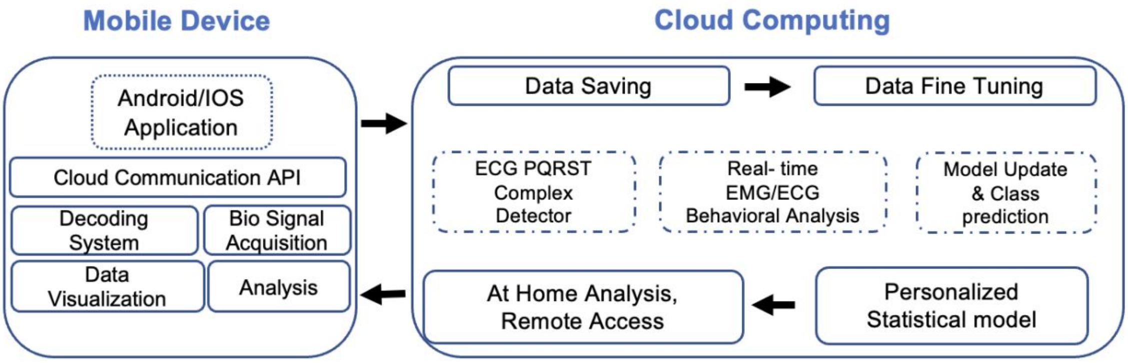

Figure S15: Flow chart for personalized at-home health monitoring.

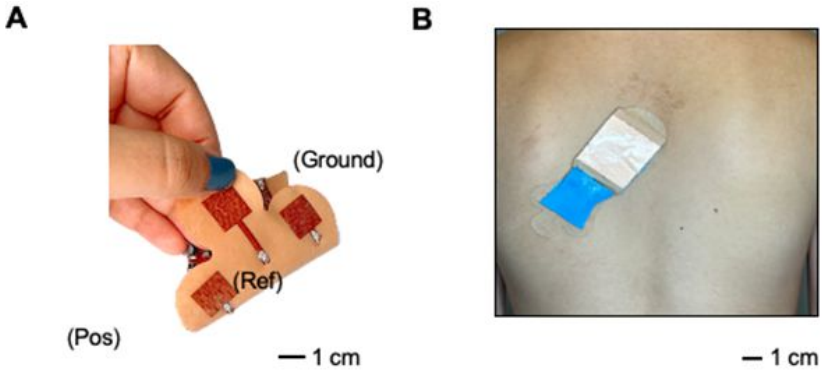

Figure S16: A) Photo of an array of electrodes; B) Photo of an integrated device mounted on the back.

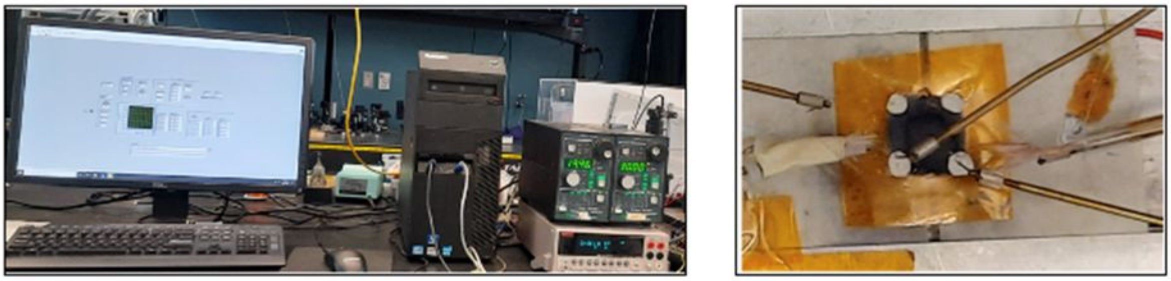

Figure S17: Photos of an experimental setup measuring thermoelectric properties (left) and CNT film on the station (right).

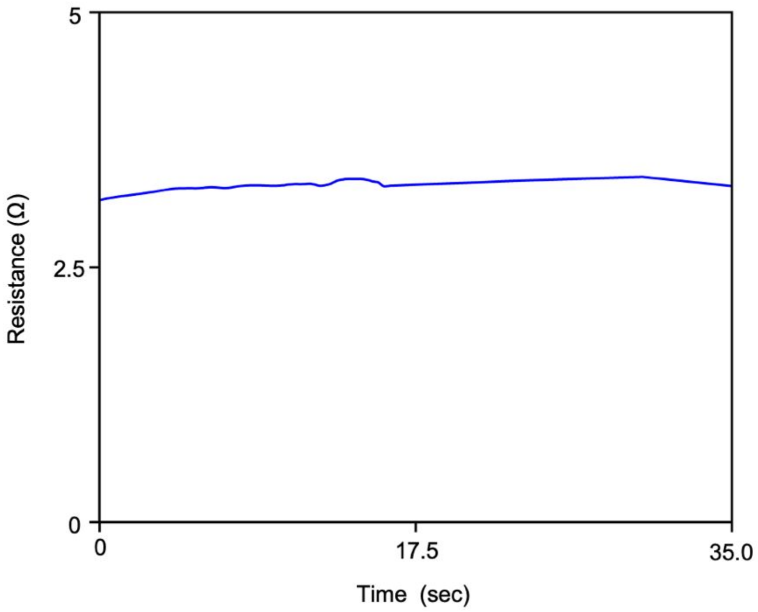

Figure S18: A) The real-time mechanical performance of the TEG device when TEG device was bent, folded, and swung in different directions, as shown in Video S1

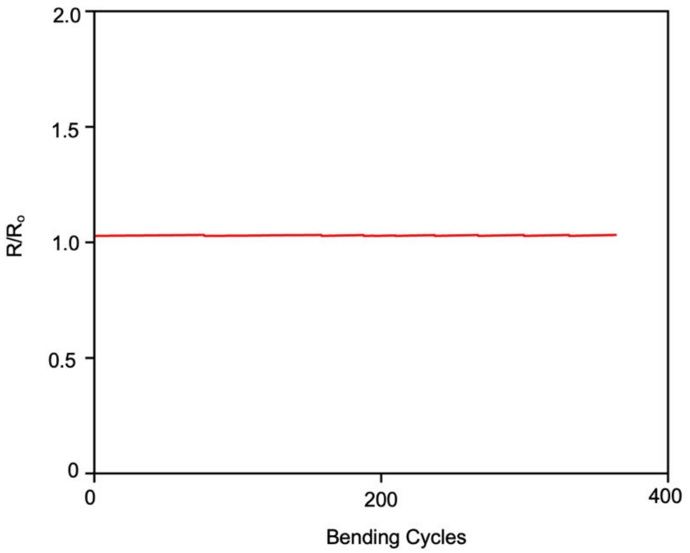

Figure S19: A) The real-time bending performance of electrodes for various bending cycles

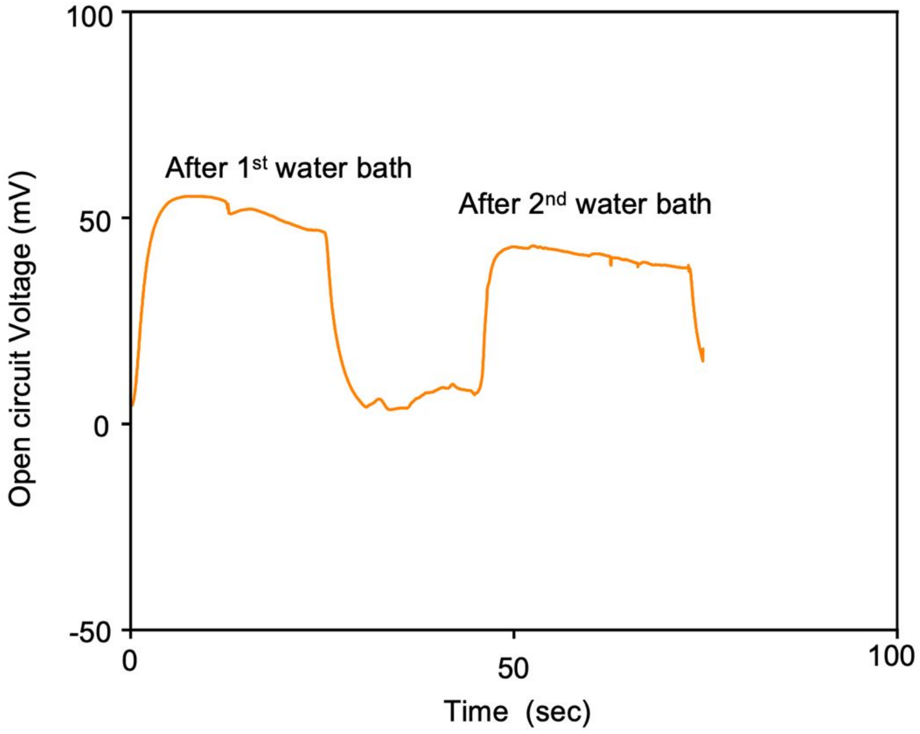

Figure S20: A) The real-time performance of the TEG device in a wet environment

Table S1: List of components for the power management circuit.

Part	Value	Device	Package	Sheet
C1	10 nF 25 V XR7	0402CAP.KW	0402-CAP	1
C2	4.7 uF 10 V XR5	CAP0603-CAP	0603-CAP	1
C3	4.7 uF 10 V XR5	CAP0603-CAP	0603-CAP	1
C4	100 nF 16 V XR5	0402CAP.KW	0402-CAP	1
R1	9.76M 1%	0402RES.KW	0402-RES	1
R2	5.90M 1%	0402RES.KW	0402-RES	1
R3	4.42M 1%	0402RES.KW	0402-RES	1
R4	5.62M 1%	0402RES.KW	0402-RES	1
R5	4.42M 1%	0402RES.KW	0402-RES	1
R6	6.12M 1%	0402RES.KW	0402-RES	1
R7	3.83M 1%	0402RES.KW	0402-RES	1
R8	3.32M 1%	0402RES.KW	0402-RES	1
R9	6.12M 1%	0402RES.KW	0402-RES	1
R10	542K 1%	0402RES.KW	0402-RES	1
R11	4K7 5%	0402RES.KW	0402-RES	1
U$1	BRC2518T220K	2012	DFE252012C	1
U$2	BC847BLP4	BC847BLP4	DFN1006	1
U$3	0402LED	GREEN	0402LED	1
U1	BQ25504	BQ25504	BQ25504	1

Table S2: List of components for the biosensor circuit.

Description	Code	Value	Part Number	Quantity
Low-Power Analog Front END			ADS1292	1
Wireless System on Chip (SoC)			nRF52832	1
32 MHz 8 pF Surface Mount (SMD) Crystal		32 MHz, 8 pF		1
32.768 kHz 9 pF SMD Crystal		32.768 kHz, 9 pF	XC1947CT-ND	1
2.4GHz Antenna		2.4 GHz		1
Balun Filter			712-1623-1-ND	1
1.8 Voltage Regulator		1.8 V		1
3.3 Voltage Regulator		3.3 V		1
1 uF Ceramic Capacitor	0402	1 uF		3
1 uF Tantalum Capacitor	0402	1uF		1
1 uF Ceramic Capacitor	0603	1 uF		1
4.7 uF Ceramic Capacitor	0402	4.7 uF	1276-1481-1-ND	1
10 uF Ceramic Capacitor	0402	10 uF		4
1 nF Ceramic Capacitor	0402	1 nF		1
4.7nF Ceramic Capacitor	0402	4.7nF		2
100 nF Ceramic Capacitor	0402	100 nF	1276-1043-1-ND	9
1 pF Ceramic Capacitor	0402	1 pF	1276-1595-1-ND	1
12 pF Ceramic Capacitor	0402	12 pF		4
100 pF Ceramic Capacitor	0402	100 pF	1276-1025-1-ND	1
1 MOhm Thick Film Resistor	0402	1 Mohm	311-1.00MLRCT-ND	1
10 kOhm Thick Film Resistor	0402	10 kOhm		3
10 uH Inductor	0603	10 uH	445-67551-ND	1
2.7 nH Inductor	0402	2.7 nH		1
3.9 nH Inductor	0402	3.9 nH		1

Video S1: Mechanical performance of flexible TEG

Video S2: Performance comparison between our device and a commercial one.

Video S3: Sustainable performance of TEG in a wet environment

Video S4: Comparison of TEG performance in different climates

Video S5: Self-powered wearable health monitoring system.

Video S6: Mechanical performance of flexible PCB.

Video S7: Device that measures EMG signals.

Video S8: Device that measures ECG signals compared to a commercial one.
